# Changes to bacterial communities and soil metabolites in an apple orchard as a legacy effect of different intercropping plants and soil management practices

**DOI:** 10.3389/fmicb.2022.956840

**Published:** 2022-08-08

**Authors:** Xiaolong Li, Yannan Chu, Yonghua Jia, Haiying Yue, Zhenhai Han, Yi Wang

**Affiliations:** ^1^College of Horticulture, China Agricultural University, Beijing, China; ^2^Institute of Horticulture, Ningxia Academy of Agricultural and forestry Sciences, Yinchuan, China

**Keywords:** bacterial community, microbiome, agricultural management, soil metabolic products, PacBio SMRT long-read sequencing

## Abstract

Intercropping is an important soil management practice for increasing orchard productivity and land-use efficiency because it has beneficial effects on soil microbial communities and soil properties. However, there is relatively little information available regarding the effects of different crops/grasses on soil microbial communities and soil metabolic products in apple orchards in arid and semi-arid regions. In this study, we showed the microbial communities of apple, intercropping plants, and sandy waste soil, using the third-generation PacBio SMRT long-read sequencing technology. Our results also revealed that the microbial communities and soil metabolic properties differed significantly between apple and the sandy waste soil and the intercropping plants. Intercropping could significantly enrich diverse microbial species, microbial nitrogen, and microbial carbon of soil. Moreover, intercropping with licorice showed better effects in recruiting beneficial microbes, compared to grass and pepper, significantly enriching species belonging to some well-known taxa with beneficial effects, including *Bacillus, Ensifer, Paenibacillus, Rhizobium*, and *Sphingomonas*. Thus, intercropping with licorice may improve apple tree growth and disease resistance. Furthermore, *Bradyrhizobium* and *Rubrobacter* were included among the keystone taxa of apple, whereas *Bacillus, Chitinophaga, Stenotrophobacter, Rubrobacter*, and *Luteimonas* were the keystone taxa of the intercropping plants. The results of our study suggest that intercropping with licorice is a viable option for increasing apple orchard productivity.

## Introduction

The arid and semi-arid regions (annual rainfall: ~200 mm) in northwestern China are ideal for cultivating fruit trees because they receive sufficient sunlight and there is a large temperature difference between day and night. However, the development of the fruit industry has been constrained by limited water resources and low soil fertility (Wang et al., 2007). Adopting suitable orchard soil management practices (e.g., tillage and intercropping) is important for improving the soil quality in apple orchards (Sun et al., 2022). Traditionally, clean tillage has been included in apple orchard management practices. However, clean tillage is conducive to the evaporation of water and organic matter loss while also decreasing the soil nutrient supply capacity (Ma et al., 2021). Intercropping is an important way to improve the soil's ecological environment, maintain soil moisture levels, and increase the soil organic matter and nutrient contents in orchards (Wu et al., 2011).

The intercropping of grass in orchards typically involves growing grass in the area between two rows of fruit trees (distance between trees: ~3.5–4.5 m), and this agroforestry system has developed rapidly in recent years (Triplett and Dick, 2008). Natural grass, clover, ryegrass, *Vulpia myuros*, and alfalfa are the most commonly used plants for intercropping in orchards (Wang et al., 2014; Coller et al., 2019; Sun et al., 2022). To increase profits, some orchard managers also grow commercial crops (e.g., chili and licorice) between rows of fruit trees (Li et al., 2022). Previous studies revealed that interplanting with *V. myuros* can suppress the growth of other weeds, improve the physicochemical properties of orchards, and provide nutrients for fruit trees (Brown and Rice, 2010). If the intercropping plant has a fibrous root system (e.g., rattan grass), it can maintain soil moisture levels, stabilize the soil structure, and inhibit soil loss in orchards (Ishii et al., 2007; Wang et al., 2011). In addition to changing the orchard's soil nutrient contents, plants used for intercropping can also alter the soil microbiota (Chen et al., 2014; Wang et al., 2020). Moreover, intercropping helps regulate the abundance of plant organic resources and mediates C and N biogeochemical cycles, which substantially affect the soil's metabolic footprint (Bever, 2015).

Soil microbial communities play an important role in the transformation of soil nutrients and organic matter, and their compositions and structure can be affected by plant species and their associated metabolic products (Wu et al., 2011). For example, when intercropping plants are introduced or invaded, the dynamic changes in the soil microbial community lead to a corresponding change in soil C accumulation (Mooshammer et al., 2014). Compared with clean tillage management, intercropping can significantly increase the diversity and abundance of microbial communities in orchard soil (Lacombe et al., 2009; Li et al., 2020). Furthermore, the plant–root microbiota may co-evolve with plant growth processes, with different plant species used for intercropping resulting in different microbial community compositions and abundance (Van Der Heijden and Wagg, 2013). Changes in root metabolic products, organic matter, and resource utilization due to the intercropping of different plants may lead to the development of specialized microbial communities that subsequently modulate plant growth (Houlden et al., 2008). For example, intercropping with ryegrass can dramatically increase the abundance of *Nitrospira* species, ascomycetes, and basidiomycetes, whereas intercropping with alfalfa or white clover may significantly increase the abundance of actinomycetes and the microbial biomass carbon content (Rodríguez-Loinaz et al., 2008). This microbial composition and structural differences may be associated with plant roots synthesizing specific amino acids, phenolic compounds, and terpenoids that attract certain microbes to colonize the surrounding soil (Kuffner et al., 2008).

The main root exudates from leguminous plants shaped the microbial diversity, structure, and functional groups related to the N cycle during SOM mineralization, and intercropping with aromatic plants increased N release in the orchard soil (Zhang et al., 2021). Recent studies have shown that the soil N and C storage are enhanced through the promotion of biological N fixation by leguminous plants (Sun et al., 2022). For example, Chinese fir plantations intercropping with leguminous plants could increase the soil N content and result in promoting the growth and diversity of soil microbial communities (Zhang et al., 2020). The improvement in the growth and diversity of soil microbial communities in the intercropping system could significantly increase productivity and abiotic and biotic stress resistance (Santonja et al., 2017; Ye et al., 2019). Hence, planting multispecies intercropping plants, such as the combination of legume (licorice) or non-legume species (natural grass), may provide additional benefits by not only increasing the abundance and diversity of beneficial microbes but also the soil nutrients such as soil N and C (Wortman et al., 2012; Wang et al., 2016b). Licorice is a deep-rooted perennial leguminous plant with high resistance to drought and cold. It has been wildly used as the intercropping plant in arid and semi-arid regions in northwestern China (Li et al., 2022). Intercropping with licorice or other non-legume species has become the most prevalent soil management practice in apple orchards. However, much of the related research has focused on metabolic differences, with relatively few studies analyzing the correlation between microbial communities and the metabolic footprint of different intercropping plants. However, how mixed intercropping plants regulate the soil microbial communities and the differences in composition, diversity, and co-occurrence network of different intercropping plants is still unclear.

In this study, we chose an apple orchard located in the middle of a sandy wasteland in Ningxia province (China), in which there were no signs of human soil reclamation activities. We examined the soil nutrient-related properties (e.g., pH and chemical elements), microbial communities, and metabolic products following three treatments (licorice, natural grass, and pepper). To accurately characterize the apple tree- and intercropping plant-associated microbial communities, we used PacBio SMRT technology for amplicon sequencing experiments. The main objectives of this study were as follows: (1) to identify the effect of different intercropping plants on the soil bacterial communities and soil metabolic profiles in the orchard system; (2) to reveal the core microbiota associated with apple trees and intercropping plant species and elucidate the effects of soil factors on microbial communities; (3) to find the diversity and composition differences between legume and non-legume intercropping plants in apple orchard soil micro-ecology.

## Materials and methods

### Sample collection and analysis of soil chemical and metabolite properties

Field experiments were performed at an apple orchard (133 hectares) in Ningxia province in northwestern China (37.9N, 105.9E). The watering and fertilizer were supplied by drip irrigation depending on the demand of apple plants. The average annual rainfall and temperature at the sample collection sites were 200.2 mm and 8.5°C, respectively (Li et al., 2022). To increase the profitability of the orchard, pepper and licorice were cultivated between some of the rows of apple trees, whereas natural grass was grown between the remaining rows of apple trees. The licorice is a perennial leguminous plant with deep roots, while the pepper and grasses are annual plants. The peppers are planted by the manager in May and natural grass was grown every year. Samples were collected in October 2021 according to a randomized selection method involving three biological replicates. Samples were collected only in 2021, enabling us to evaluate microbiome changes after 7 years (2015–2021) of continued planting of these three intercropping plants in a stable environment. In total, 9 sampling sites were selected, with five biological replicates collected per site. When collecting soil samples, the surface soil was removed, and the soil was dug out with part of the plant roots. The root soil samples were collected by manually shaking the root, and the collected root soil samples were placed in sterile plastic zip-lock bags. All the root soil samples were transported back to the laboratory in an icebox and immediately stored at −80°C for the subsequent DNA extraction. The soil physiochemical properties of total carbon (TOC), organic carbon, total nitrogen (TN), and ammonium nitrate were measured using an elementar vario TOC analyser (Elementar, Hanau, Germany) (Ichihashi et al., 2020). The available phosphorus (AP) and potassium were measured using an elementar analyser (Vario EL III, Elementar, Hanau, Germany) (Neilsen et al., 2014). The microbial biomass N (MBN) and microbial biomass C (MBC) were determined according to the standard protocol described by Vance et al. (1987) with the extraction of N and C from unfumigated and fumigated soils. An extraction efficiency coefficient value of 0.38 and 0.45 was used to convert the difference in C and N between fumigated and unfumigated samples in MBC and MBN, respectively. All the measured soil chemical properties are listed in Supplementary Table 1.

### Extraction of DNA from root microbiome samples

Total microbial genomic DNA was extracted from 0.5 g of fresh soil using the FastDNA Spin Kit for Soil DNA Extraction (MP Biomedicals, USA). The quality of the extracted DNA was checked by 1% agarose gel electrophoresis, whereas the DNA concentration was determined using the NanoDrop 2000c UV-Vis spectrophotometer (Thermo Scientific, Wilmington, USA). All DNA samples were stored at −80°C. For each sample, the entire 16S rRNA gene was amplified by PCR using the primer pair 27F (5′-AGAGTTTGATCCTGGCTCAG-3′) and 1492R (5′-TACCTTGTTACGACTT-3′) and the following PCR program: 95°C for 3 min; 30 cycles of 95°C for 30 s, 56°C for 30 s, and 72°C for 3 min; 72°C for 10 min; hold at 4°C. The PCR amplification of each sample was performed in triplicate to minimize the stochastic effect. The purified PCR products were pooled in equimolar amounts and then used to construct the amplicon library for the SMRT sequencing analysis using the PacBio sequencing platform (Novogene Co., Ltd., Beijing, China).

### 16S rRNA gene bioinformatic and phylogenetic analyses

The circular consensus sequencing (CCS) reads were obtained from the raw sequence data using the pbccs (v4.02) software, with -min-passes = 5 (https://github.com/PacificBiosciences/ccs). The data were converted to the fastq format using the BAM2fastx tool (https://github.com/pacificbiosciences/bam2fastx/). The CCS reads were demultiplexed according to the barcode-primer sequences using flexbar (Dodt et al., 2012), with the barcode trimmed, and a 0.1 mismatch rate. The CCS reads with a full-length primer were removed to avoid unwanted multi-primer artifacts (Tedersoo et al., 2018). The CCS reads were filtered for quality using the USEARCH 11 software (Edgar, 2018). The chimeric sequences were removed using the *de novo* and reference-based (i.e., RDP Gold database as a reference) (Edgar, 2016) methods of the VSEARCH software (Rognes et al., 2016). The remaining non-chimeric reads were dereplicated, sorted, and assembled into operational taxonomic units (OTUs), with singletons (<8 sequences) eliminated using the UPARSE algorithm of USEARCH 11. The taxonomic assignment of representative sequences of bacterial OTUs was performed using the SINTAX algorithm and the SILVA 16S rRNA database (release 138), with a confidence threshold of 0.8.

### Diversity analyses

Diversity analyses were performed using the R 4.0 (R Core Team) and several R packages including vegan (Oksanen et al., 2016), pheatmap, dplyr, igraph (Csardi and Tamas, 2006), and other packages in the R environment were employed for the data analysis unless otherwise stated. The original OTU table was rarefied to 5,267 reads (the fewest bacterial reads among samples) to enable equal comparisons before analyzing alpha-diversity and beta-diversity. The alpha-diversity (i.e., Shannon and ACE indices) among compartment niches and treatments was analyzed using the “diversity” and “estimate” functions in the vegan package (Oksanen et al., 2016). An independent one-way analysis of variance (ANOVA) followed by Tukey's honestly significant difference test at the 5% significance level was used for comparing the alpha-diversity and taxonomic differences between groups. The edgeR package was used to identify significant OTUs enriched specifically in the root-soil of apples or root-soil of different intercropping plants. All the samples were normalized using the trimmed mean of M-values (TMM) normalization method and a False Discovery Rate (FDR) corrected value of *p* <0.05 (Robinson et al., 2010). Additionally, differential abundance analysis between three intercropping plants (glass, pepper, and glycyrrhiza) was generated using the mean values of relative abundance (>0.2% threshold, transformed by log2) using the limma package in R and visualized using ternary plots (Bulgarelli et al., 2015). The linear discriminant analysis effect size (LEfSe) software was used to analyze the statistically significant differential abundance of microbial taxa corresponding to sandy waste soil, root-soil of apples, and root-soil of intercropping plants (Paulson et al., 2013). The distance matrix of the bacterial community was constructed by calculating the Bray–Curtis dissimilarity and plotted according to an unconstrained principal coordinate analysis (PCoA). Significant differences in the beta-diversity among treatments and groups were determined on the basis of a permutational multivariate ANOVA (PERMANOVA), which was performed using the “adonis” function (Oksanen et al., 2016). A distance-based redundancy analysis (RDA) was used to assess the influence of environmental factors on the bacterial community structure (Oksanen et al., 2016). Pearson's product-moment correlation-based Mantel test was used to determine the correlation between the overall variation in the bacterial communities and each environmental factor.

### Phylogenetic tree of core microbial taxa

The bacterial OTUs common to more than 90% of the samples in the same group were used to define the core microbial taxa. A total of 136 bacterial OTUs were included in the core taxa among samples. The OTU-associated representative sequences were extracted and used for constructing maximum likelihood phylogenetic trees (5,000 ultrafast bootstrap replicates and 1,000 replicates of the SH-like approximate likelihood ratio test) using the IQ-TREE software (Nguyen et al., 2015). The core OTUs used to construct phylogenetic trees are listed in Supplementary Table 2. The representative sequences of the core OTUs were aligned using MUSCLE software (Edgar, 2004) and then each aligned sequence was trimmed using the default parameters of the trimAL software (Capella-Gutiérrez et al., 2009). On the basis of all trimmed sequences, the phylogenetic model was selected using the ModelFinder software. The best-fit model according to the BIC algorithm was used for constructing maximum likelihood phylogenetic trees (Nguyen et al., 2015). The phylogenetic trees were made and annotated with the relative abundance of each core taxa among groups according to the procedure described by Qi et al. (2022) using the online open-source tool Interactive Tree of Life (iTOL) (Letunic and Bork, 2019).

### Co-occurrence network construction and analysis

Co-occurrence networks of microbial communities were constructed based on high abundant OTUs for root-soil of apple and root-soil of intercropping plants. Significant Spearman rank correlations between the OTUs of different plant species were calculated to construct co-occurrence networks. Only the significant and robust (correlation values < −0.7 or > 0.7 and *p* < 0.001) correlations were retained for the downstream analysis. The networks were visualized using the Fruchterman-Reingold layout algorithm (10^4^ permutations) of the igraph package (Csardi and Tamas, 2006). Furthermore, the “greedy optimisation of modularity” algorithm was used to identify co-occurrence network modules, which comprise a nodal substructure with a higher density of edges within groups than between groups (Hartman et al., 2018). Separate co-occurrence networks were constructed according to the different compartments of the network's topological characteristics. The topological characteristics representing the connectivity and complexity of the co-occurrence networks, including the number of edges, modules, and nodes as well as the density and modularity, were calculated using the igraph package (Csardi and Tamas, 2006). The keystone taxa are important for plant health and contribute to plant growth and resistance to biotic factors (Wei et al., 2019; Zhou et al., 2021). Therefore, the keystone taxa were identified for each network using the NetShift online platform (https://web.rniapps.net/netshift/) (Kuntal et al., 2019).

### Metabolite extraction, profiling, and correlation with microbial communities

For each sample, 0.6 ml of 2-chlorophenylalanine (4 ppm) in methanol (−20°C) was added to ~200 mg of lyophilized soil in sample tubes, which were then vortexed for 30 s. Sterile glass beads (100 mg) were added to the tubes before grinding the samples using a high-throughput tissue homogenizer with 60 Hz for 90 s (SCIENTZ-48, China). The ground samples underwent a 30-min ultrasonication treatment. They were then incubated in an ice bath for 30 min before being centrifuged at 12,000 × *g* for 10 min. An ~300 μl aliquot of the supernatant was filtered through a 0.22 μm membrane to obtain the processed samples for the LC-MS analysis (Seybold et al., 2020). The Thermo Vanquish system equipped with an ACQUITY UPLC^®^ HSS T3 column (150 × 2.1 mm, 1.8 μm; Waters) was used for the chromatographic separation. The column was maintained at 40°C, whereas the autosampler was kept at 8°C. The gradient elution of analytes was conducted using 0.1% formic acid in water (mobile phases A2), 0.1% formic acid in acetonitrile (mobile phases B2) or 5 mM ammonium formate in water (mobile phases A3), and acetonitrile (B3), at a flow rate of 0.25 ml/min. For each sample, ~2 μl was injected after an equilibration step. The increasing linear gradient of solvent B2/B3 (v/v) was as follows: 0–1 min, B2/B3 (2%); 1–9 min, B2/B3 (2–50%); 9–12 min, B2/B3 (50–98%); 12–13.5 min, B2/B3 (98%); 13.5–14 min, B2/B3 (98–2%); and 14–20 min, B2 (positive model) or 14–17 min, B3 (2%, negative model). The ESI-MSn analysis was conducted using the Thermo Q Exactive mass spectrometer, with a spray voltage of −2.5 and 3.5 kV in the negative and positive modes, respectively. The sheath gas and auxiliary gas were set at 30 arbitrary units and 10 arbitrary units, respectively. Additionally, the capillary temperature was set at 325°C. The analyzer was scanned at a mass resolution of 70,000 over a mass range (m/z) of 81–1,000 for a full scan. Data-dependent acquisition MS/MS treatments were performed using an HCD scan, with a normalized collision energy of 30 eV. Correlation between taxa abundances at the phyla and genera level and metabolite profiles were tested only for differentially abundant bacterial genera (P_adj_ <0.05) using Spearman's ρ, respectively (Best and Roberts, 1975). Differences in metabolite profiles and bacterial genera in different samples were tested by two-way ANOVA, repeated measurements with the Bonferroni *post hoc* test, and the significantly correlated bacterial genera and metabolite profiles were visualized by heatmap graphs.

### Data availability statement

The raw read data described herein have been deposited into the Genome Sequence Archive (accession number CRA006577) of the National Genomics Data Center (https://ngdc.cncb.ac.cn/gsa) of the China National Center for Bioinformation at the Beijing Institute of Genomics, Chinese Academy of Sciences.

## Results

### Microbial community compositions after different treatments

**Figure 1:**
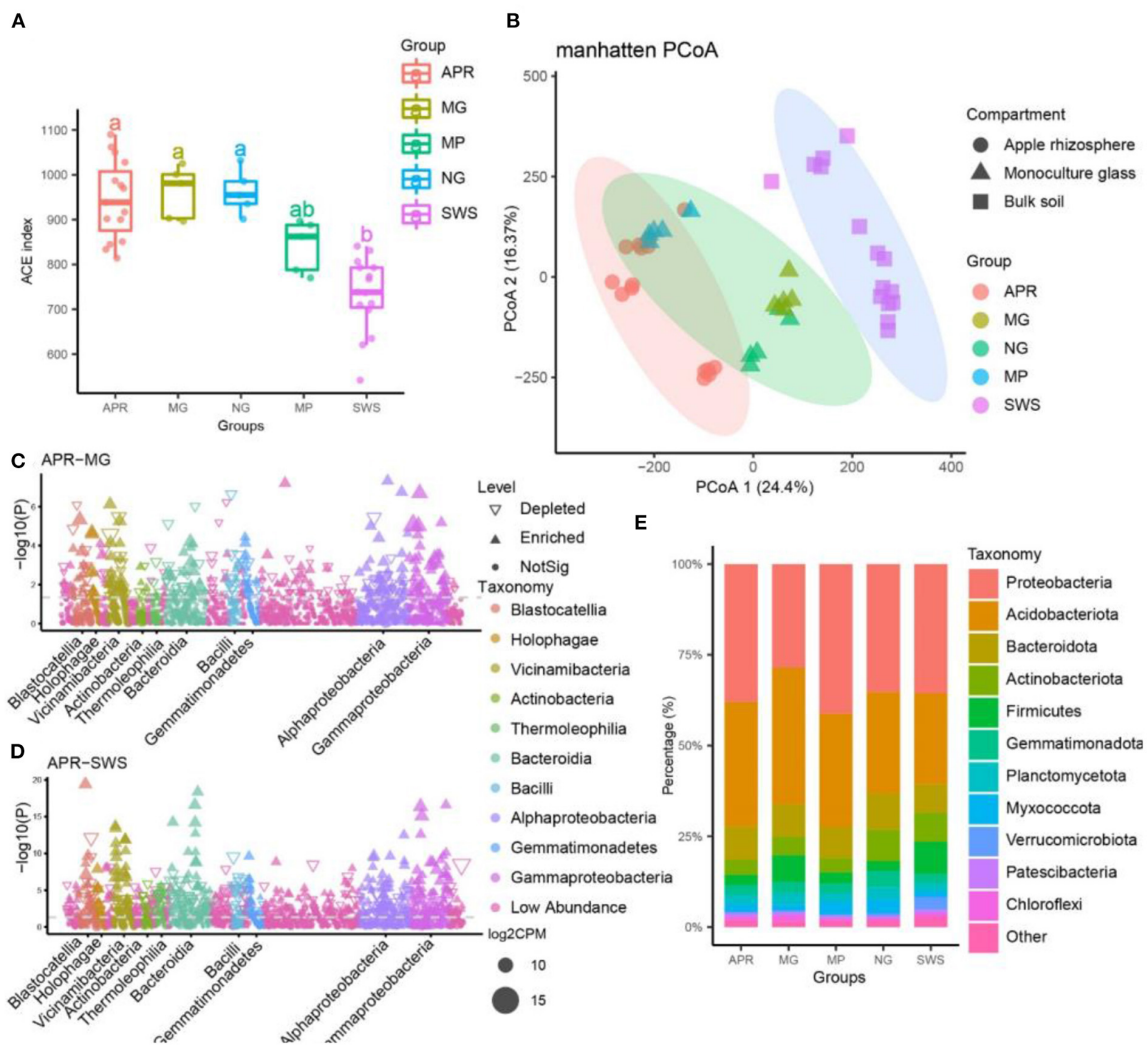
Microbial diversity of apple, intercropping plants, and sandy waste soil (SWS). **(A)** Microbial diversity of different groups according to the ACE index. The lines inside the boxes represent the median values (95% confidence). The significant differences in the alpha indices between groups are indicated by different lowercase letters (*p* < 0.05). **(B)** Manhattan distance analysis of bacterial communities from different samples (principal coordinates PCo1 and PCo2). The microbiomes of apple, intercropping plants and SWS were significantly separated from each other along the two axes (*P* < 0.001, permutational multivariate analysis of variance, PERMANOVA). The ellipses cover 90% of each group. **(C,D)** Manhattan plots present significantly enriched bacterial operational taxonomic units (OTUs) in the apple vs. licorice **(C)** and apple vs. SWS **(D)** comparisons. **(E)** Relative abundance of the predominant bacteria (at the phylum level) in different samples. APR, root soil of apple plant; MG, root soil of licorice; NG, root soil of natural grass; MP, root soil of pepper plant; SWS, soil of sandy waste.

After filtering the raw sequencing data, 343,238 high-quality CCS reads (7,628 reads per sample) generated by the PacBio SMRT sequencing platform were retained. The bacterial reads were assembled into 1,665 OTUs with 100% sequence identity using the UNOISE algorithm. The rarefaction curve of each group indicated that bacterial diversity curves nearly reached the asymptote, implying our sequencing depths were sufficient for most of the samples (Supplementary Figure 1). Comparative bacterial diversity analyses were performed after the sequence numbers were normalized to the lowest sequencing depths (5,267 reads per sample). A comparison of the alpha-diversity (ACE index) indicated that the bacterial diversity was significantly higher for apple and the intercropping plants (grass, licorice, and pepper) than for the sandy waste soil (SWS) (Tukey's honestly significant difference test, *P* < 0.05) (Figure 1A). Accordingly, the long-term cultivation of plants can significantly increase soil microbial diversity. Additionally, the bacterial diversity was significantly higher in samples of grass and licorice compared to those of pepper samples, implying grass and licorice can enhance microbial diversity better than pepper (Tukey's honestly significant difference test, *P* < 0.05) (Figure 1A). Apple, grass, and licorice did not significantly affect bacterial alpha-diversity, which was similar for all three species (Figure 1A). However, the PCoA analysis revealed that the bacterial communities affected by the treatments according to Manhattan dissimilarities, indicated that 40.77% of the total variation was explained by the first two axes. Based on the adonis analysis, the bacterial community of apple varied significantly from the bacterial communities of SWS, grass, and licorice, indicating the microbial communities of apple and the intercropping plants differed from the microbial community of SWS (PERMANOVA, *P* < 0.001; Figure 1B and Supplementary Table 3). Furthermore, the microbial community of apples was clustered with that of pepper (PERMANOVA, *P* > 0.05), but the microbial compositions were significantly separated from those of licorice and grass (PERMANOVA, *P* < 0.001; Figure 1B, and Supplementary Table 3). Hence, the bacterial communities were strongly associated with different plant species, and orchard management practices and intercropping species significantly influence the soil microbial community.

### Microbial taxonomy and identification of enriched taxa in response to apple and intercropping plants

The bacterial OTUs were assigned to 30 known bacterial phyla, 76 classes, 160 orders, 186 families, and 327 genera. More specifically, Proteobacteria (22.8%), Acidobacteriota (16.6%), Planctomycetota (10.6%), Patescibacteria (9.1%), Actinobacteriota (8.0%), Gemmatimonadota (5.3%), Myxococcota (4.4%), Firmicutes (3.9%), Verrucomicrobiota (2.6%), Chloroflexi (2.3%), and Armatimonadota (1.0%) were the predominant phyla (Figure 1E). At the family level, Chitinophagaceae (6.4%), Gemmatimonadaceae (3.8%), Pirellulaceae (2.6%), Vicinamibacteraceae (2.5%), Vicinamibacterales_uncultured (2.4%), Sphingomonadaceae (2.3%), Blastocatellaceae (2.2%), Bacilaceae (2.1%), Pyrinomonadaceae (2.0%), and Comamonadaceae (1.6%) were the most common taxa. Moreover, about 22% of all sequences were not assigned to any known family, indicative of a considerable abundance of novel bacteria in the apple orchard soil. A total of 228, 154, 258, and 464 bacterial OTUs were significantly more enriched in the apple group than in the licorice, pepper, grass, and SWS groups, respectively (Figures 1C,D and Supplementary Figure 2, Supplementary Table 4). These results indicate a large number of specific bacteria were recruited by apple trees (Supplementary Figure 2 and Supplementary Table 4). In contrast, 149, 88, 250, and 378 bacterial OTUs were, respectively, more enriched in the licorice, pepper, grass, and SWS groups than in the apple group (Supplementary Figure 2 and Supplementary Table 4).

**Figure 2:**
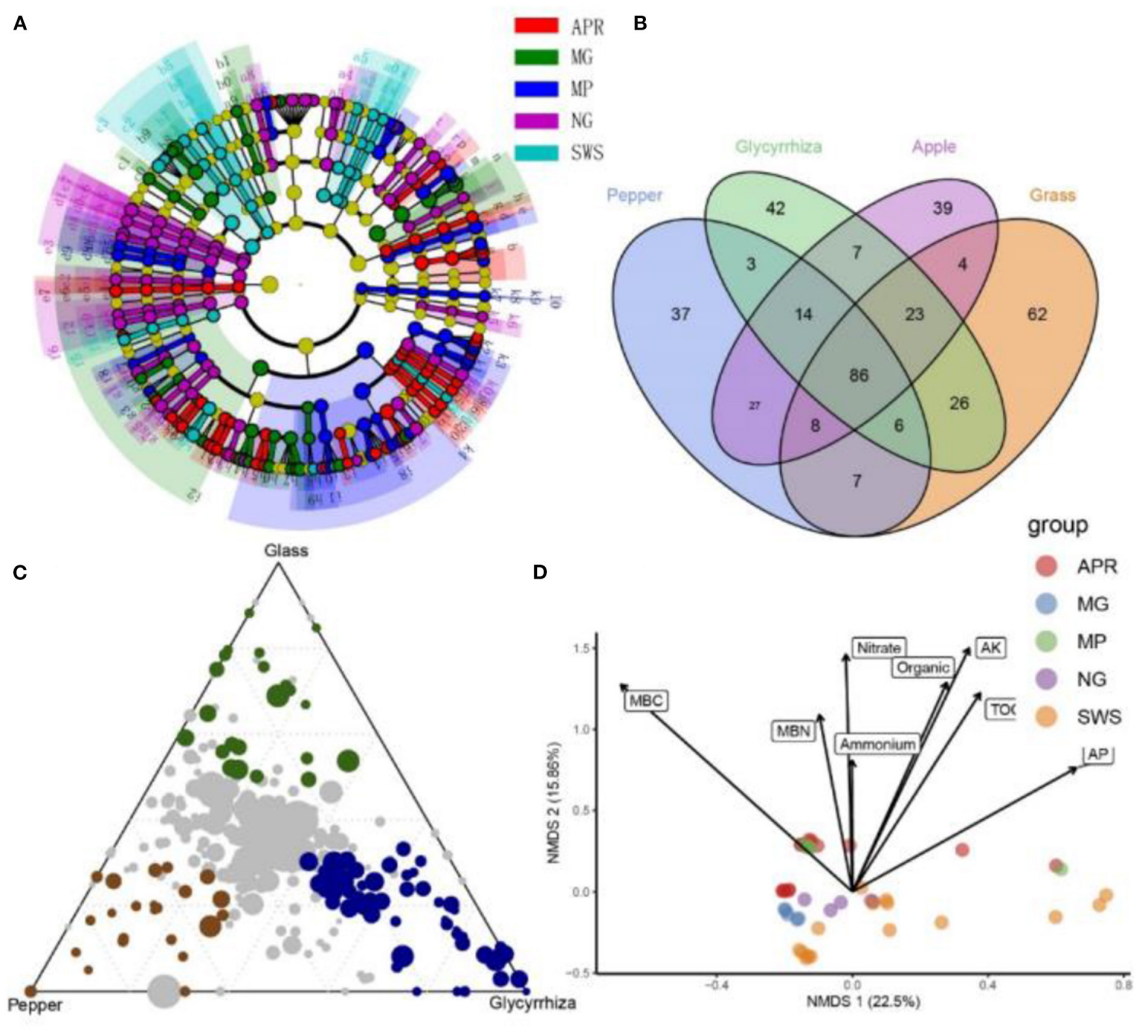
Shared and significantly enriched microbial taxa among groups. **(A)** The linear discriminant analysis effect size (LefSe) plot presents the LDA effect size taxonomic cladogram of APR, MG, MP, NG, and SWS. Significantly discriminant microbial taxa are colored according to the highest-ranked group for that taxon. Indicator microbial taxa with LDA > 3.5, FD > 2, *p* < 0.05 in bacterial communities associated with different groups were presented in the graph. **(B)** Venn diagrams presenting the shared and specific OTUs in different root-soil samples, including root soil of apple, root-soil of grass, root-soil of glycyrrhiza, and root-soil of pepper. **(C)** The ternary plot presents significantly enriched bacterial OTUs in natural grass (green filled circles), pepper (brown filled circles), and licorice (blue filled circles). The gray dots in the center of the ternary graph represent non-significant bacterial OTUs shared by all groups. **(D)** The redundancy analysis (RDA) ordination of significant soil properties in root-soil samples and SWS samples. Vectors show fitted values of soil factors significantly correlated within ordination space. The correlations between the soil factors and RDA axes are represented by the length and angle of the arrows.

We also conducted a LEfSe analysis of the indicator species to identify specific taxa among groups. For example, the bacteria belonging to the following taxa were specifically enriched in the apple group: *Arenimonas, Acidibacter, Bauldia, Comamonas, Hyphomicrobium, Pedomicrobium, Methyloceanibacter, Novosphingobium, Nitrospira*, Nitrosomonadaceae, Nitrospiraceae, *Nitrospiria*, Blastocatellaceae, *Terrimonas*, Hyphomicrobiaceae, *Stenotrophomonas*, and *Xylophilus* (Figure 2A and Supplementary Figure 3). Thus, these bacteria may be included in the core taxa of apple. Moreover, we observed that the intercropping plants recruited different bacterial species, resulting in diverse community compositions (Figures 1B, 2A). Venn diagrams revealed 86 bacterial OTUs that were shared by apple and the intercropping plants (Figure 2B). Compared with pepper (37 OTUs), grass (62 OTUs), and licorice (42 OTUs) had more group-specific OTUs. The limma and LEfSe analyses further revealed the differences in the enriched bacterial taxa among intercropping plants (Figure 2C and Supplementary Table 5). Compared with the pepper group, the grass group was significantly enriched with species belonging to *Altererythrobacter, Blastococcus*, Bryobacter, *Chitinophaga, Cystobacter, Domibacillus, Dongia, Ferruginibacter, Flavitalea, Flavisolibacter*, Gemmatimonadaceae, *Kocuria, Lautropia, Massilia, Methylomirabilia, Mesorhizobium*, Myxococcaceae, *Myxococcus*, Microscillaceae, Oxalobacteraceae, *Phycisphaerae, Steroidobacter*, Steroidobacteraceae, and *Solirubrobacter* (Figures 2A,C, Supplementary Figure 3, and Supplementary Table 5). Furthermore, the species belonging to *Bacillus, Bradyrhizobium, Ensifer, Fictibacillus, Hirschia*, Hyphomonadaceae, Rhizobiaceae, *Rhodoplanes, Rubrobacter, Paenibacillus*, Paenibacillaceae, *Pararhizobium, Puia, Rhizobium, Sphingomonas, Vicinamibacter*, and *Vicinamibacteria* were significantly more enriched in the licorice group than in the other groups (Figures 2A,C, Supplementary Figure 3, and Supplementary Table 5). The results of the RDA and Mantel test were used to characterize the significant factors that influenced the distribution and composition of the microbial communities. Approximately, 19.01% of the total variance in the bacterial community composition could be explained by the analyzed environmental factors (Figure 2D). Specifically, the soil chemical properties TOC [variance explained (VE) = 4.82%, *P* = 0.001], nitrate (VE = 4.28%, *P* = 0.001), available potassium (VE = 2.79%, *P* = 0.001), microbial carbon (MBC) (VE = 3.92%, *P* = 0.001), and AP (VE = 3.35%, *P* = 0.051) were the most important factors affecting the bacterial composition, followed by ammonium (VE = 3.35%, *P* = 0.05) and organic matter (VE = 2.89%, *P* = 0.05) (Supplementary Table 6). The Mantel test results indicated that nitrate and MBC were strongly correlated with the microbial communities of the apple, intercropping plant, and SWS samples (Supplementary Table 6). Moreover, when exogenous carbon and nutrient resources were not applied, the MBC and microbial nitrogen contents were significantly higher for the intercropping plants than for the SWS (Supplementary Figure 8).

### Identification of the core OTUs in response to apple and intercropping plants

**Figure 3:**
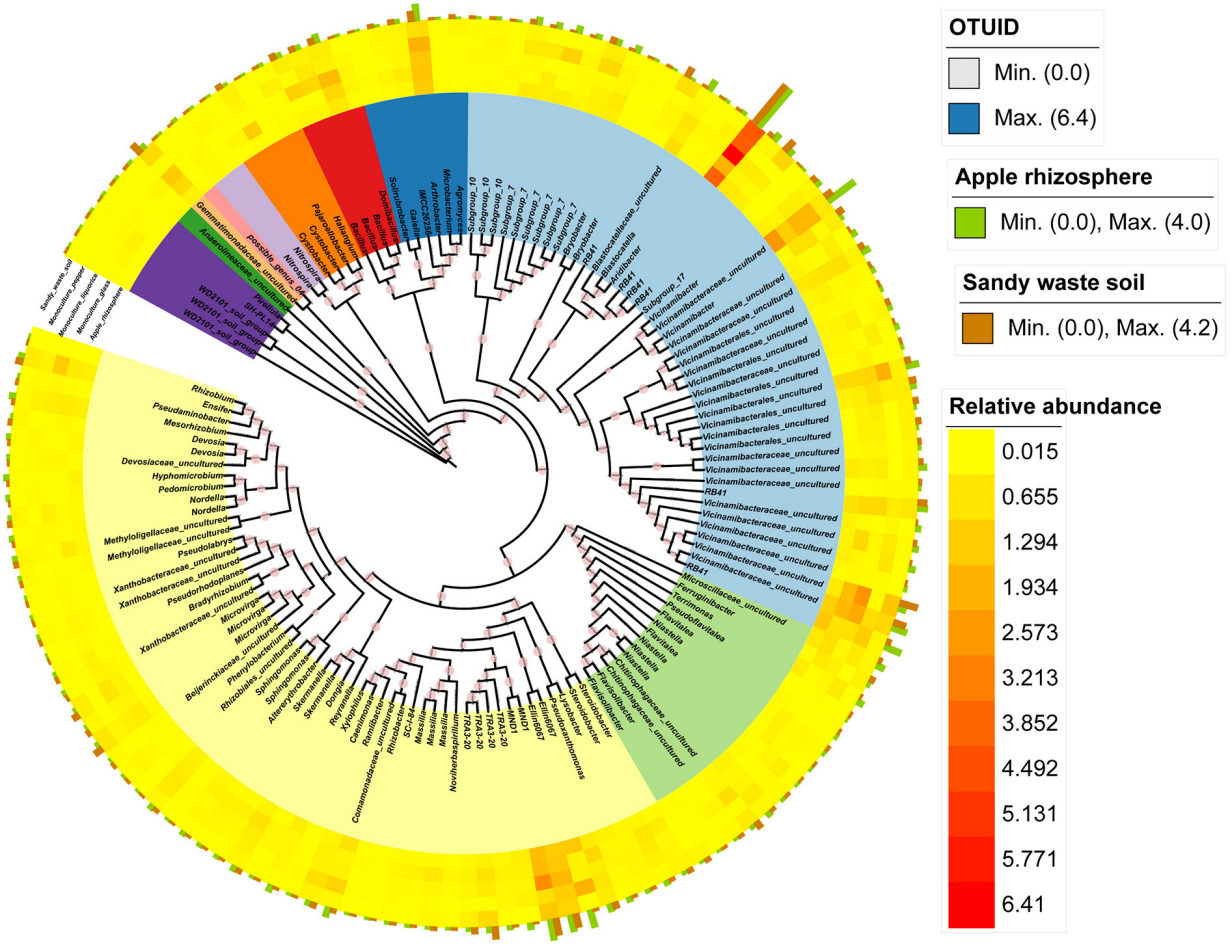
Phylogenetic relationships among the core bacterial OTUs (present in >90% of the analyzed samples). The annotated heatmap presents the relative abundance of core OTUs in different samples. The green bar plot presents the abundant core OTUs in apple, whereas the brown bar plot presents the abundant core OTUs in SWS.

**Figure 4:**
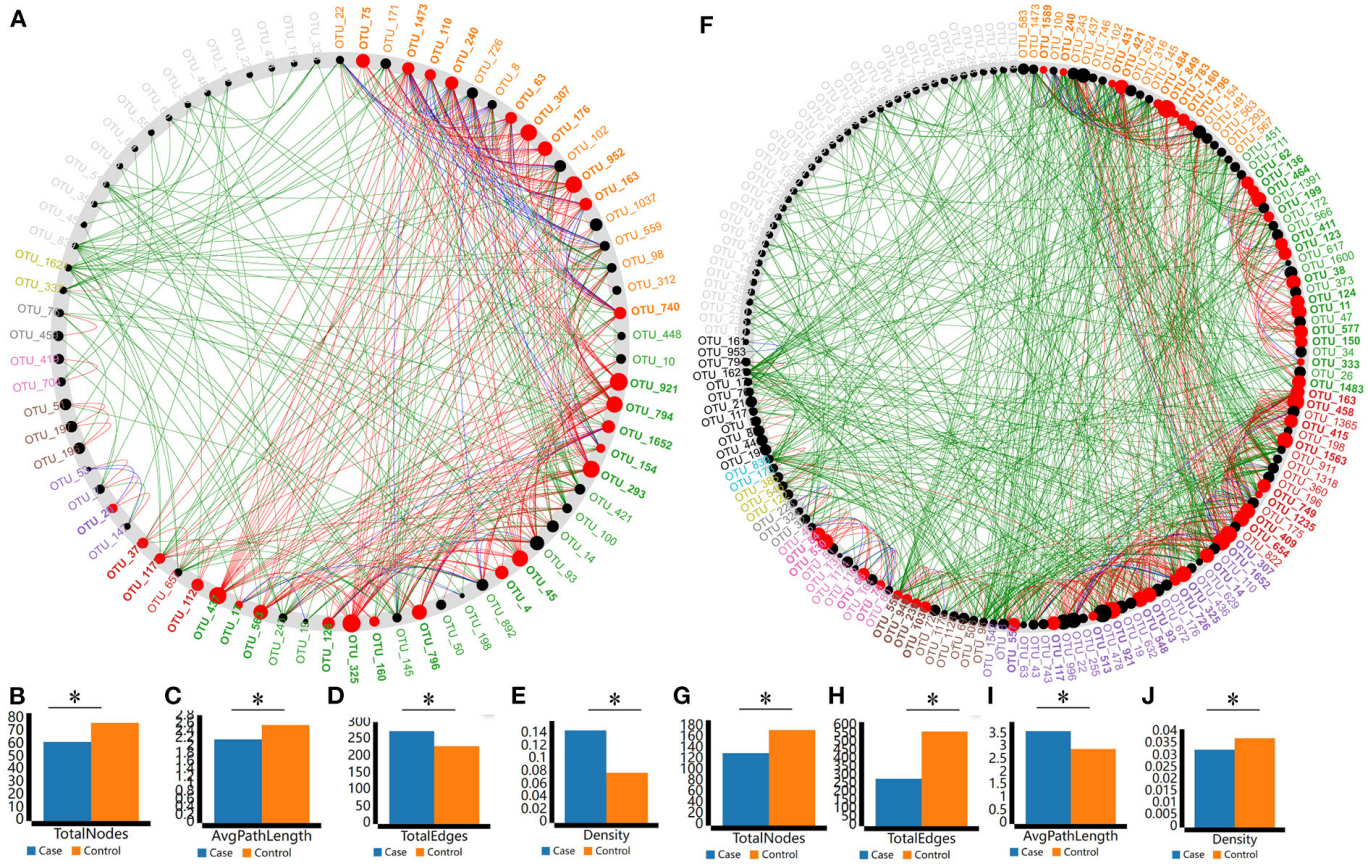
The microbial correlations were inferred from OTUs abundance profiles using the Spearman method and only the robust and significant (correlation values < −0.7 or > 0.7 and *P* < 0.001) correlations were kept for the construction of co-occurrence networks. Potential keystone taxa on the basis of co-occurrence network analyses of microbial communities. Node sizes and colors are proportional to their scaled NESH score (i.e., score revealing important microbial taxa of microbial association networks). The large red nodes in each plot represent particularly important keystone taxa of apple **(A)** and the intercropping plants **(F)**. Each node corresponds to the bacterial OTUs, line colors represent the node (taxa) connections as follows: association present only in apple **(A)**/intercropping plant **(F)** microbiomes (red edges), association present only in intercropping microbiomes (green edges), and association present in both apple **(A)**/intercropping plant **(F)** and SWS microbiomes (blue edges). Bar plots illustrate the comparisons of the total number of nodes **(B)**, average path lengths **(C)**, the total number of edges **(D)**, and the network density **(E)** between apple and SWS. The comparisons between apple and intercropping plants were illustrated by bar plots of the total number of nodes **(G)**, average path lengths **(H)**, the total number of edges **(I)**, and the network density **(J)**. *P < 0.05.

We identified the core microbial species in the apple orchard on the basis of the bacterial OTUs present in more than 90% of the samples. The core taxa of the apple orchard included 136 bacterial OTUs (Figures 4A,B). The core bacterial taxa in the apple orchard included 75 genera (Supplementary Table 2), of which *Arthrobacter, Aridibacter, Bacillus*, Gemmatimonadaceae_uncultured, *Terrimonas*, Blastocatellaceae_uncultured, *Terrimonas, RB41, MND1*, Microscillaceae_uncultured, *Vicinamibacter*, and Vicinamibacteraceae_uncultured were the dominant genera in the apple group (among all core microbes) (Figure 3). In contrast, Comamonadaceae_uncultured, *Ensifer, Devosia, Dongia, Hyphomicrobium, Steroidobacter, Terrimonas, Mesorhizobium, RB41*, and Vicinamibacteraceae_uncultured were the most common taxa in SWS (among all core microbes) (Figure 3).

The co-occurrence networks revealed significant differences among apple, the intercropping plants, and SWS (Supplementary Figures 4a–c). The apple microbiome networks were much more complex than the SWS microbiome networks, with a longer average path length (Figure 4B), more nodes and edges (Figures 4C,D), and higher modularity (Figure 4E and Supplementary Table 7). The comparison of the microbiome networks of apples and the intercropping plants indicated that the total number of nodes (Figure 4G) and edges (Figure 4H) and the network density (Figure 4J) were significantly greater for apples than for the intercropping plants, whereas the average path length was significantly greater for the intercropping plants than for apples (Figure 4I). These findings suggest that the microbiome networks of apples and the intercropping plants facilitated more interactions between diverse species and were more stable than the SWS microbiome networks. Next, the NetShift analysis was performed to identify the keystone taxa in the co-occurrence networks of different groups. The keystone microbial species are initial microbiomes for plant health and could potentially help plants gain resistance to biotic and abiotic stresses. The NetShift results showed that 18 different bacterial taxa were identified as the keystone taxa of apple plants (Figure 4A and Supplementary Table 8). In addition, 15 bacterial taxa were detected as the keystone taxa of the intercropping plants (Figure 4F and Supplementary Table 8). Some well-reported beneficial bacteria including *Bradyrhizobium, Bacillus*, Chitinophaga, *Rubrobacter*, and *Stenotrophobacter* were the keystone taxa of intercropping plants.

### Metabolic and functional profiles of bacterial communities in response to group niches

**Figure 5:**
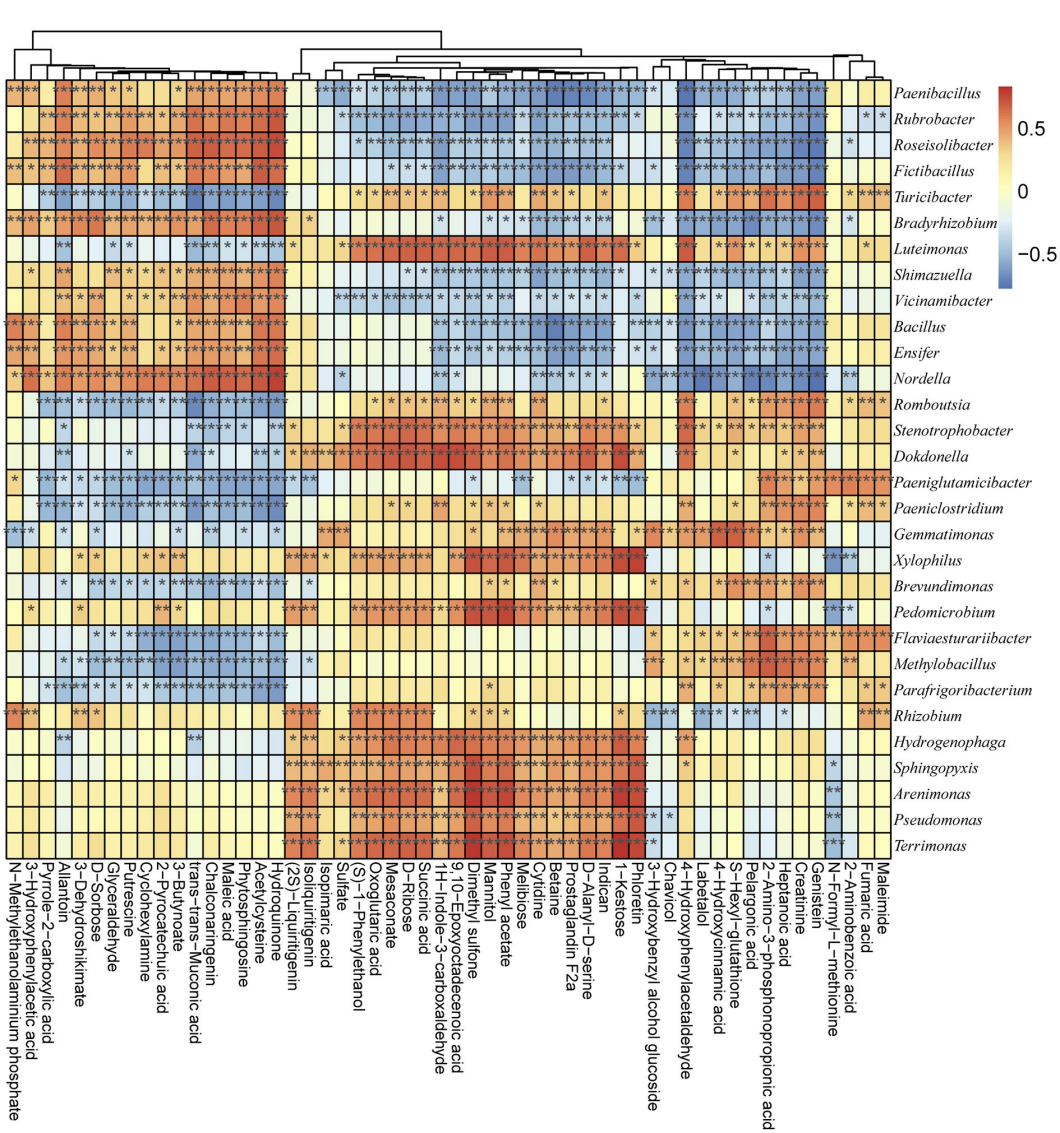
Associations between metabolic products and microbial communities according to Spearman's correlation algorithm. The horizontal axis presents the metabolic products, whereas the vertical axis presents the microbial communities at the genus level. Red and blue indicate positive and negative correlations, respectively. *P < 0.05 and |R| > 0.7, **P < 0.01 and |R| > 0.7, and ***P < 0.001 and |R| > 0.7.

The bacterial community metabolic and functional differences between groups were detected *via* GC-MS and PICRUSt analyses (according to COG and KO). According to the secondary metabolites' annotation, a total of 54 metabolic profiles were detected from all samples (Supplementary Table 9). Among the secondary metabolites, the Hydroquinone, Maleic acid, Creatinine, 3–Hydroxybenzyl alcohol glucoside, D–Ribose, Phloretin, Pelargonic acid, Maleimide, and 4–Hydroxycinnamic acid were the most abundant metabolic profiles (Supplementary Figure 5). The functional classification detected the following as the main enriched KEGG pathways among the examined bacterial communities: ABC transporters, amino acid related enzymes, bacterial motility proteins, DNA repair and recombination proteins, two-component system, purine metabolism, secretion system, oxidative phosphorylation, peptidases, transcription factors, and pyrimidine metabolism (Supplementary Figure 6). Thus, the metabolic activities in the soil were closely related to the microbial communities. Spearman's correlation algorithm revealed a significant correlation (|R| > 0.70, *P* < 0.05) between the metabolic products and the dominant genera of the microbial communities in different groups (Figure 5). Using a threshold of |R| > 0.70 and *p* < 0.05, 46 bacterial genera were determined to be significantly correlated with the soil metabolic properties (Figure 5 and Supplementary Table 10). Among the bacterial genera, *Arenimonas, Dokdonella, Nordella, MND1, Pedomicrobium, Puia, Pedomicrobium, Xylophilus*, and *Tumebacillus* were the key taxa highly related to soil properties (Supplementary Table 10). For example, *Arenimonas* was significantly and positively correlated with mannitol (*R* = 0.7079, *P* < 0.05), phloretin (*R* = 0.74040, *P* < 0.05), phenylacetate (*R* = 0.7562, *P* < 0.05), 1-kestose (*R* = 0.7914, *P* < 0.05), and dimethyl sulfone (*R* = 0.8015, *P* < 0.05). Additionally, succinic acid was significantly positively correlated with *IS-44* (*R* = 0.7070, *P* < 0.05) and Reyranellaceae_uncultured (*R* = 0.7350, *P* < 0.05) but negatively correlated with Solirubrobacteraceae_uncultured (*R* = −0.7282, *P* < 0.05). Epoxyoctadecenoic acid was positively correlated with *Dokdonella* (*R* = 0.7426, *P* < 0.05), Reyranellaceae_uncultured (*R* = 0.7877, *P* < 0.05), and Rhodanobacteraceae_uncultured (*R* = 0.7235, *P* < 0.05). Trans-trans-muconic acid was negatively correlated with *Clostridium sensu stricto* (*R* = −0.7562, *P* < 0.05) and *Turicibacter* (*R* = −0.7212, *P* < 0.05). An analysis of the correlations between metabolic products and microbial communities at the phylum level (Supplementary Figure 7) indicated that Firmicutes was significantly correlated with betaine (*R* = −0.8187, *P* < 0.05), melibiose (*R* = −0.7814), prostaglandin F2a (*R* = −0.7789, *P* < 0.05), D-alanyl-D-serine (*R* = −0.7661, *P* < 0.05), indican (*R* = −0.7329, *P* < 0.05), and N-methylethanolaminium phosphate (*R* = 0.7257, *P* < 0.05) (Supplementary Figure 7 and Supplementary Table 11). Methylomirabilota was correlated with genistein (*R* = −0.7336, *P* < 0.05), 4-hydroxyphenylacetaldehyde (*R* = −0.7300, *P* < 0.05), 1H-indole-3-carboxaldehyde (*R* = −0.7070, *P* < 0.05), acetylcysteine (*R* =0.7328, *P* < 0.05), hydroquinone (*R* = 0.7458, *P* < 0.05), and trans-muconic acid (*R* = 0.7480, *P* < 0.05) (Supplementary Figure 7 and Supplementary Table 11).

## Discussion

In this study, we characterized the microbial communities associated with apple and intercropping plants in an orchard and the SWS on the basis of PacBio SMRT sequencing data. Previous studies revealed that intercropping can enhance soil properties, including organic matter and nutrient contents, thereby promoting plant growth and increasing the productivity of orchards (Palviainen et al., 2005; Qiao et al., 2014). Moreover, intercropping can modulate soil physicochemical properties, which influence soil microbial community compositions and diversity (Francioli et al., 2016). In the present study, the soil chemical properties TOC, nitrate, available potassium, AP, and ammonium were significantly correlated with bacterial communities. Substantial amounts of intercropping plant residues (e.g., roots and leaves) can transform the orchard soil, with positive effects on the soil organic matter, ultimately leading to increased soil fertility (Neilsen et al., 2014). For example, licorice is a perennial leguminous plant species that can recruit root-associated rhizobia that fix nitrogen and increase the soil TN, alkali-hydrolyzed nitrogen, and organic matter contents. Moreover, intercropping plants can facilitate the propagation of the soil microbiome to diversify the soil microbial community and recruit beneficial microbes that will promote plant growth and disease resistance (Sulkava and Huhta, 1998; Lacombe et al., 2009; Li et al., 2020). The results of our study were consistent with the findings of these earlier investigations that, the intercropping plants were beneficial to microbial diversity and promoted the stability and nutrient enrichment of the soil ecosystem (Bever, 2015). More specifically, we demonstrated that all of the examined intercropping plants significantly increased the apple orchard soil microbial diversity. However, the influence of different intercropping plants and the induced changes in metabolic products will need to be more precisely characterized.

In the present study, the plant types significantly affected the microbial abundance and compositions and the soil metabolic properties. For example, compared with the effects of pepper, intercropping with licorice and grass resulted in a more diverse bacterial community. The licorice is a perennial legume species plant, whereas pepper is an annual plant species in the family Solanaceae. A highly developed root system that produces specific soil metabolic compounds may be associated with the enrichment of highly diverse microbes (Li et al., 2022). Moreover, the intercropping plants may be a source of various nutrients and carbon compounds including vitamins, nucleic acids, and amino acids, which may help recruit diverse microbes (Liu et al., 2013; Qiao et al., 2014). For example, previous studies have demonstrated that legume intercropping plants in the orchard could promote the diversity of the microbial community and enhance the abundance of beneficial soil microbes (Bever, 2015). The results of the current study provide further evidence that intercropping plants, such as licorice and grass, can increase soil microbial abundance and biomass and the co-occurrence network parameter of the microbial community. These results are consistent with previous research, suggesting that increases in the abundance and diversity of microbial communities can lead to more efficient substrate decomposition and accelerated nutrient release. The increase in soil nutrient contents could significantly enhance plant growth and disease resistance (Wang et al., 2016a).

Owing to the close relationship between host plants and microbes, the development of specific plant traits may be induced by increasing the soil microbial diversity (Reinhold-Hurek et al., 2015; Santhanam et al., 2015). Batten et al. revealed that after introducing intercropping plants *Aegilops triuncialis* and *Centaurea solstitialis* to native plants, the number of sulfate-reducing microbes, sulfur oxidizing microbes, and arbuscular mycorrhizal fungi significantly increased (Batten et al., 2006). Moreover, more diverse intercropping species could improve the soil nutrient cycle and improve fruit quality by regulating microbial community complexity and stability (Sun et al., 2022). Our findings demonstrated that different intercropping plants can significantly diversify the soil microbiome. For example, compared with grass and pepper, intercropping with licorice was better able to enrich the species belonging to *Bacillus, Bradyrhizobium, Ensifer, Fictibacillus, Hirschia*, Hyphomonadaceae, Rhizobiaceae, *Rhodoplanes, Rubrobacter, Paenibacillus*, Paenibacillaceae, *Pararhizobium, Puia, Rhizobium, Sphingomonas, Vicinamibacter*, and *Vicinamibacteria*. These licorice-enriched microbes include many well-known beneficial species. Specifically, *Bacillus, Ensifer, Paenibacillus, Rhizobium*, and *Sphingomonas* species can promote plant growth and increase nutrient availability by inducing plants to secrete auxin while also fixing nitrogen and decomposing organic P and organic matter (Son et al., 2009; Desai et al., 2016; Asaf et al., 2020; Zhou et al., 2020). In addition to their plant growth-promoting effects, *Bacillus, Paenibacillus, Rhizobium*, and *Sphingomonas* species can protect plants from phytopathogens and abiotic stresses (e.g., drought and salinity) by synthesizing lipopeptide-type compounds that induce plant systemic resistance and the production of amino acids, proline, soluble sugars, and exopolysaccharides (Patel et al., 2015a,b; Asaf et al., 2020). The intercropping plants are also helpful for maintaining the stability and ecological functions of microbial communities by recruiting and restructuring soil microbiota (Zhao et al., 2022). These findings imply that intercropping with licorice can increase the resistance of apple trees to pathogens and enhance tree growth. Our NetShift analysis characterized several keystone taxa of apple and the intercropping plants. According to taxonomic annotations, the keystone taxa of apple included *Bradyrhizobium, Rubrobacter*, and many unidentified bacterial taxa. The keystone species among the intercropping plants included *Bacillus, Chitinophaga, Stenotrophobacter, Rubrobacter, Luteimonas*, and several unidentified bacterial taxa. These keystone microbes often had higher relative abundances and greater connections with other species, which may significantly impact network stability. The intercropping plants' enriched keystone microbes could potentially play important roles in plant growth and stress resistance (Wei et al., 2019). Considered together, these results suggest that many novel microbes may play important roles in influencing the health of apple and intercropping plants. Future related research should focus on isolating these novel keystone taxa and evaluating their functions and interactions with host plants. Overall, our study generated relevant information for improving and implementing agricultural management practices that will increase the productivity of apples and land-use efficiency.

## Data availability statement

The datasets presented in this study can be found in online repositories. The names of the repository/repositories and accession number(s) can be found in the article/Supplementary material.

## Author contributions

ZHH and YW planned and supervised this research. XLL performed the field/wet-lab experiments, conducted the data analysis, visualization, and writing. YNC, YHJ, and HYY contributed to the data analysis and revision of the manuscript. All authors read and approved the final manuscript.

## Funding

This work was financially supported by the Key R&D Project of Autonomous Region (2021BBF02014 and 2022BBF02035), the Independent Innovation Special Project of the Academy of Agricultural Sciences (NGSB-2021-1), and the Earmarked Fund for the China Agriculture Research System (CARS-27).

## Conflict of interest

The authors declare that the research was conducted in the absence of any commercial or financial relationships that could be construed as a potential conflict of interest.

## Publisher's note

All claims expressed in this article are solely those of the authors and do not necessarily represent those of their affiliated organizations, or those of the publisher, the editors and the reviewers. Any product that may be evaluated in this article, or claim that may be made by its manufacturer, is not guaranteed or endorsed by the publisher.

## Supplementary material

Supplementary Figure 1 Rarefaction curves of bacterial groups from apple, licorice, grass, pepper, and SWS.

Supplementary Figure 2 **(a–d)** Manhattan plots presenting significantly enriched bacterial OTUs in the apple vs licorice **(a)**, apple vs pepper **(b)**, apple vs grass **(c)**, and apple vs SWS **(d)** comparisons.

Supplementary Figure 3 LDA effect size taxonomic cladogram of different groups (The graphics in Figure_and_Table).

Supplementary Figure 4 Visualization of the co-occurrence networks of apple, intercropping plant, and SWS microbial communities. **(a)** Co-occurrence networks of apple samples. **(b)** Co-occurrence networks of intercropping plant samples. **(c)** Co-occurrence networks of SWS samples. Nodes represent individual OTUs, with the bacterial phyla indicated by different colors. Links between nodes indicate significant correlations between OTUs.

Supplementary Figure 5 Predominant secondary metabolic profiles in the apple, licorice, pepper, grass, and SWS groups.

Supplementary Figure 6 Predominant KEGG pathways in the apple, licorice, pepper, grass, and SWS groups.

Supplementary Figure 7 Associations between metabolic products and bacterial communities according to Spearman's correlation algorithm. The horizontal axis presents the metabolic products, whereas the vertical axis presents the microbial communities at the phylum level. Red and blue indicate positive and negative correlations, respectively. ^*^*P* < 0.05 and |R| > 0.7.

Supplementary Figure 8 Microbial nitrogen (MBN) **(a)** and microbial carbon (MBC) **(b)** contents in apple, licorice, pepper, grass, and SWS groups. The lines inside each box represent the median values (95% confidence). Significant differences in the MBN and MBC contents between groups are indicated by different lowercase letters (*P* < 0.05).

Supplementary Table 1 Soil properties of each sample.

Supplementary Table 2 Taxonomic information for the core bacterial OTUs shared by more than 90% of all samples.

Supplementary Table 3 Microbial community differences among groups as revealed by PERMANOVA.

Supplementary Table 4 Differential abundance analysis of bacterial enrichment patterns (FDR-adjusted *P* < 0.05) in the apple *vs* pepper, apple *vs* licorice, and apple *vs* grass comparisons.

Supplementary Table 5 Significantly enriched bacterial OTUs in the apple, pepper, and licorice samples.

Supplementary Table 6 Results of the distance-based RDA for exploring the variance in the microbial communities explained by the soil properties. *, **, and *** indicate statistical significance at *P* < 0.05, *P* < 0.01, and *P* < 0.001, respectively.

Supplementary Table 7 Topological properties of the apple, intercropping plant, and SWS co-occurrence networks.

Supplementary Table 8 Taxonomy information of keystone taxa associated with root-soil of apple plants and intercropping plants.

Supplementary Table 9 Secondary metabolic profiles of root-soil of apple plants, intercropping plants, and SWS.

Supplementary Table 10 Correlations between soil properties and microbial taxa at the genus level.

Supplementary Table 11 Correlations between soil properties and microbial taxa at the phylum level.

## References

[B1] Asaf S. Numan M. Khan A. L. Al-Harrasi A. (2020). *Sphingomonas*: from diversity and genomics to functional role in environmental remediation and plant growth. Crit. Rev. Biotechnol. 40, 138–152. 10.1080/07388551.2019.1709793 31906737

[B2] Batten K. M. Scow K. M. Davies K. F. Harrison S. P. (2006). Two invasive plants alter soil microbial community composition in serpentine grasslands. Biol. Invasions 8, 217–230. 10.1007/s10530-004-3856-8

[B3] Best D. J. Roberts D. E. (1975). Algorithm AS 89: the upper tail probabilities of Spearman's rho. J. R. Stat. Soc. Ser. C. 24, 377–379. 10.2307/2347111

[B4] Bever J. D (2015). Preferential allocation, physio-evolutionary feedbacks, and the stability and environmental patterns of mutualism between plants and their root symbionts. New Phytol. 205, 1503–1514. 10.1111/nph.13239 25561086

[B5] Brown C. S. Rice K. J. (2010). Effects of belowground resource use comlementarity on invasion of constructed grassland plant communities. Biol. Invasions 12, 1319–1334. 10.1007/s10530-009-9549-6

[B6] Bulgarelli D. Garrido-Oter R. Muench P. C. Weiman A. Droege J. Pan Y.. (2015). Structure and function of the bacterial root microbiota in wild and domesticated barley. Cell Host Microbe 17, 392–403. 10.1016/j.chom.2015.01.011 25732064 PMC4362959

[B7] Capella-Gutiérrez S. Silla-Martínez J. M. Gabaldón T. (2009). trimAl: a tool for automated alignment trimming in large-scale phylogenetic analyses. Bioinformatics 25, 1972–1973. 10.1093/bioinformatics/btp348 19505945 PMC2712344

[B8] Chen Y. Wen X. Sun Y. Zhang J. Wu W. Liao Y. (2014). Mulching practices altered soil bacterial community structure and improved orchard productivity and apple quality after five growing seasons. Sci. Hortic. 172, 248–257. 10.1016/j.scienta.2014.04.010

[B9] Coller E. Cestaro A. Zanzotti R. Bertoldi D. Pindo M. Larger S.. (2019). Microbiome of vineyard soils is shaped by geography and management. Microbiome 7, 1–15. 10.1186/s40168-019-0758-7 31699155 PMC6839268

[B10] Csardi G. Tamas N. (2006). The igraph software package for complex network research. *Interjournal Complex Syst*. 1695. Available online at: http://igraph.sf.net

[B11] Desai S. Kumar G. P. Amalraj L. D. Bagyaraj D. J. Ashwin R. (2016). “Exploiting PGPR and AMF biodiversity for plant health management,” in Microbial Inoculants in Sustainable Agricultural Productivity, eds D. P. Singh, H. B. Singh, and R. Prabha (Springer), 145–160.

[B12] Dodt M. Roehr J. T. Ahmed R. Dieterich C. (2012). FLEXBAR—flexible barcode and adapter processing for next-generation sequencing platforms. Biology 1, 895–905. 10.3390/biology1030895 24832523 PMC4009805

[B13] Edgar R. C (2004). MUSCLE: multiple sequence alignment with high accuracy and high throughput. Nucleic Acids Res. 32, 1792–1797. 10.1093/nar/gkh340 15034147 PMC390337

[B14] Edgar R. C (2016). UCHIME2: improved chimera prediction for amplicon sequencing. BioRxiv [Preprint]. 10.1101/074252

[B15] Edgar R. C (2018). Accuracy of taxonomy prediction for 16S rRNA and fungal ITS sequences. PeerJ. 6:e4652. 10.7717/peerj.4652 29682424 PMC5910792

[B16] Francioli D. Schulz E. Lentendu G. Wubet T. Buscot F. Reitz T. (2016). Mineral vs. organic amendments: microbial community structure, activity and abundance of agriculturally relevant microbes are driven by long-term fertilization strategies. Front. Microbiol. 7:1446. 10.3389/fmicb.2016.01446 27683576 PMC5022044

[B17] Hartman K. van der Heijden M. G. A. Wittwer R. A. Banerjee S. Walser J.-C. Schlaeppi K. (2018). Correction to: cropping practices manipulate abundance patterns of root and soil microbiome members paving the way to smart farming. Microbiome 6:74. 10.1186/s40168-018-0456-x 29690923 PMC5913793

[B18] Houlden A. Timms-Wilson T. M. Day M. J. Bailey M. J. (2008). Influence of plant developmental stage on microbial community structure and activity in the rhizosphere of three field crops. FEMS Microbiol. Ecol. 65, 193–201. 10.1111/j.1574-6941.2008.00535.x 18616582

[B19] Ichihashi Y. Date Y. Shino A. Shimizu T. Shibata A. Kumaishi K.. (2020). Multi-omics analysis on an agroecosystem reveals the significant role of organic nitrogen to increase agricultural crop yield. Proc. Nat. Acad. Sci. 117, 14552–14560. 10.1073/pnas.1917259117 32513689 PMC7321985

[B20] Ishii T. Matsumura A. Horii S. Motosugi H. Cruz A. F. (2007). Network establishment of arbuscular mycorrhizal hyphae in the rhizospheres between citrus rootstocks and Paspalum notatum or Vulpia myuros grown in sand substrate. Biol. Fertil. Soils 44, 217–222. 10.1007/s00374-007-0197-7

[B21] Kuffner M. Puschenreiter M. Wieshammer G. Gorfer M. Sessitsch A. (2008). Rhizosphere bacteria affect growth and metal uptake of heavy metal accumulating willows. Plant Soil 304, 35–44. 10.1007/s11104-007-9517-9

[B22] Kuntal B. K. Chandrakar P. Sadhu S. Mande S. S. (2019). 'NetShift': a methodology for understanding 'driver microbes' from healthy and disease microbiome datasets. ISME J. 13, 442–454. 10.1038/s41396-018-0291-x 30287886 PMC6331612

[B23] Lacombe S. Bradley R. L. Hamel C. Beaulieu C. (2009). Do tree-based intercropping systems increase the diversity and stability of soil microbial communities? Agric. Ecosyst. Environ. 131, 25–31. 10.1016/j.agee.2008.08.010

[B24] Letunic I. Bork P. (2019). Interactive Tree Of Life (iTOL) v4: recent updates and new developments. Nucleic Acids Res. 47, W256–W259. 10.1093/nar/gkz239 30931475 PMC6602468

[B25] Li N. Gao D. Zhou X. Chen S. Li C. Wu F. (2020). Intercropping with potato-onion enhanced the soil microbial diversity of tomato. Microorganisms 8:834. 10.3390/microorganisms8060834 32498315 PMC7357159

[B26] Li X. Yue H. Chu Y. Jia Y. Tian J. (2022). The planting of licorice increased soil microbial diversity and affected the growth and development of apple trees. Commun. Soil Sci. Plant Anal. 53, 1113–1125. 10.1080/00103624.2022.2043342

[B27] Liu Z. Lin Y. Lu H. Ding M. Tan Y. Xu S.. (2013). Maintenance of a living understory enhances soil carbon sequestration in subtropical orchards. PLoS ONE 8:e76950. 10.1371/journal.pone.0076950 24116188 PMC3792872

[B28] Ma W. Yang Z. Hou S. Ma Q. Liang L. Wang G.. (2021). Effects of living cover on the soil microbial communities and ecosystem functions of hazelnut orchards. Front. Plant Sci. 12:445. 10.3389/fpls.2021.652493 33841481 PMC8033216

[B29] Mooshammer M. Wanek W. Zechmeister Boltenstern S. Richter A. (2014). Stoichiometric imbalances between terrestrial decomposer communities and their resources: mechanisms and implications of microbial adaptations to their resources. Front. Microbiol. 5:22. 10.3389/fmicb.2014.00022 24550895 PMC3910245

[B30] Neilsen G. Forge T. Angers D. Neilsen D. Hogue E. (2014). Suitable orchard floor management strategies in organic apple orchards that augment soil organic matter and maintain tree performance. Plant Soil 378, 325–335. 10.1007/s11104-014-2034-8

[B31] Nguyen L. T. Schmidt H. A. von Haeseler A. Minh B. Q. (2015). IQ-TREE: a fast and effective stochastic algorithm for estimating maximum-likelihood phylogenies. Mol. Biol. Evol. 32, 268–274. 10.1093/molbev/msu300 25371430 PMC4271533

[B32] Oksanen J. Blanchet F. G. Friendly M. Kindt R. Legendre P. McGlinn D.. (2016). vegan: Community Ecology Package.

[B33] Palviainen M. Finér L. Mannerkoski H. Piirainen S. Starr M. (2005). Responses of ground vegetation species to clear-cutting in a boreal forest: aboveground biomass and nutrient contents during the first 7 years. Ecol. Res. 20, 652–660. 10.1007/s11284-005-0078-1

[B34] Patel R. R. Patel D. D. Thakor P. Patel B. Thakkar V. R. (2015b). Alleviation of salt stress in germination of *Vigna radiata* L. by two halotolerant *Bacilli* sp. isolated from saline habitats of Gujarat. Plant Growth Regul. 76, 51–60. 10.1007/s10725-014-0008-8

[B35] Patel R. R. Thakkar V. R. Subramanian B. R. (2015a). A *Pseudomonas guariconensis* strain capable of promoting growth and controlling collar rot disease in *Arachis hypogaea* L. Plant Soil. 390, 369–381. 10.1007/s11104-015-2436-2

[B36] Paulson J. N. Stine O. C. Bravo H. C. Pop M. (2013). Differential abundance analysis for microbial marker-gene surveys. Nat. Methods 10, 1200–1202. 10.1038/nmeth.2658 24076764 PMC4010126

[B37] Qi Z. Zhou X. Tian L. Zhang H. Cai L. Tang F. (2022). Distribution of mycotoxin-producing fungi across major rice production areas of China. Food Control. 134:108572. 10.1016/j.foodcont.2021.108572

[B38] Qiao Y. Miao S. Silva L. C. R. Horwath W. R. (2014). Understory species regulate litter decomposition and accumulation of C and N in forest soils: a long-term dual-isotope experiment. For. Ecol. Manage. 329, 318–327. 10.1016/j.foreco.2014.04.025

[B39] Reinhold-Hurek B. Bünger W. Burbano C. S. Sabale M. Hurek T. (2015). Roots shaping their microbiome: global hotspots for microbial activity. Annu. Rev. Phytopathol. 53, 403–424. 10.1146/annurev-phyto-082712-102342 26243728

[B40] Robinson M. D. McCarthy D. J. Smyth G. K. (2010). edgeR: a Bioconductor package for differential expression analysis of digital gene expression data. Bioinformatics 26, 139–140. 10.1093/bioinformatics/btp616 19910308 PMC2796818

[B41] Rodríguez-Loinaz G. Onaindia M. Amezaga I. Mijangos I. Garbisu C. (2008). Relationship between vegetation diversity and soil functional diversity in native mixed-oak forests. Soil Biol. Biochem. 40, 49–60. 10.1016/j.soilbio.2007.04.015

[B42] Rognes T. Flouri T. Nichols B. Quince C. Mahé F. (2016). VSEARCH: a versatile open source tool for metagenomics. PeerJ. 4:e2584. 10.7717/peerj.2584 27781170 PMC5075697

[B43] Santhanam R. Luu V. T. Weinhold A. Goldberg J. Oh Y. Baldwin I. T. (2015). Native root-associated bacteria rescue a plant from a sudden-wilt disease that emerged during continuous cropping. Proc. Nat. Acad. Sci. 112, E5013–5020. 10.1073/pnas.1505765112 26305938 PMC4568709

[B44] Santonja M. Rancon A. Fromin N. Baldy V. Hättenschwiler S. Fernandez C.. (2017). Plant litter diversity increases microbial abundance, fungal diversity, and carbon and nitrogen cycling in a Mediterranean shrubland. Soil Biol. Biochem. 111, 124–134. 10.1016/j.soilbio.2017.04.006

[B45] Seybold H. Demetrowitsch T. J. Hassani M. A. Szymczak S. Reim E. Haueisen J.. (2020). A fungal pathogen induces systemic susceptibility and systemic shifts in wheat metabolome and microbiome composition. Nat. Commun. 11:1910. 10.1038/s41467-020-15633-x 32313046 PMC7171108

[B46] Son S. H. Khan Z. Kim S. G. Kim Y. H. (2009). Plant growth-promoting rhizobacteria, *Paenibacillus polymyxa* and *Paenibacillus lentimorbus* suppress disease complex caused by root-knot nematode and fusarium wilt fungus. J. Appl. Microbiol. 107, 524–532. 10.1111/j.1365-2672.2009.04238.x 19457027

[B47] Sulkava P. Huhta V. (1998). Habitat patchiness affects decomposition and faunal diversity: a microcosm experiment on forest floor. Oecologia 116, 390–396. 10.1007/s004420050602 28308071

[B48] Sun Y Chen L. Zhang S. Miao Y. Zhang Y. Li Z.. (2022). Plant interaction patterns shape the soil microbial community and nutrient cycling in different intercropping scenarios of aromatic plant species. Front. Microbiol. 13:888789. 10.3389/fmicb.2022.888789 35711748 PMC9197114

[B49] Tedersoo L. Tooming-Klunderud A. Anslan S. (2018). PacBio metabarcoding of fungi and other eukaryotes: errors, biases and perspectives. New Phytol. 217, 1370–1385. 10.1111/nph.14776 28906012

[B50] Triplett G. B. Jr. Dick W. A. (2008). No-tillage crop production: a revolution in agriculture! Agron. J. 100, S153–S165. 10.2134/agronj2007.0005c

[B51] Van Der Heijden M. G. A. Wagg C. (2013). Soil microbial diversity and agro-ecosystem functioning. Plant Soil 363, 1–5. 10.1007/s11104-012-1545-4

[B52] Vance E. D. Brookes P. C. Jenkinson D. S. (1987). An extraction method for measuring soil microbial biomass C. Soil Biol. Biochem. 19, 703–707. 10.1016/0038-0717(87)90052-6

[B53] Wang P. Wang Y. Wu Q. S. (2016a). Effects of soil tillage and planting grass on arbuscular mycorrhizal fungal propagules and soil properties in citrus orchards in southeast China. Soil Tillage Res. 155, 54–61. 10.1016/j.still.2015.07.009

[B54] Wang Q. Han S. Zhang L. Zhang D. van der Werf W. Evers J. B.. (2016b). Density responses and spatial distribution of cotton yield and yield components in jujube (*Zizyphus jujube*)/cotton (*Gossypium hirsutum*) agroforestry. Eur. J. Agron. 79, 58–65. 10.1016/j.eja.2016.05.009

[B55] Wang X. B. Cai D. X. Hoogmoed W. B. Oenema O. Perdok U. D. (2007). Developments in conservation tillage in rainfed regions of North China. Soil Tillage Res. 93, 239–250. 10.1016/j.still.2006.05.005

[B56] Wang Y. Huang Q. Liu C. Ding Y. Liu L. Tian Y.. (2020). Mulching practices alter soil microbial functional diversity and benefit to soil quality in orchards on the Loess Plateau. J. Environ. Manage. 271:110985. 10.1016/j.jenvman.2020.110985 32579532

[B57] Wang Y. Zhang B. Lin L. Zepp H. (2011). Agroforestry system reduces subsurface lateral flow and nitrate loss in Jiangxi Province, China. Agric. Ecosyst. Environ. 140, 441–453. 10.1016/j.agee.2011.01.007

[B58] Wang Y. F. Shao L. L. Liu Y. X. Lv H. H. Chen Q. F. Liao M.. (2014). Effects of inter planting grass on soil organic carbon and active components of carbon pool in peach orchard. Acta. Ecol. Sin. 34, 6002–6010.

[B59] Wei Z. Gu Y. Friman V. P. Kowalchuk G. A. Xu Y. Shen Q.. (2019). Initial soil microbiome composition and functioning predetermine future plant health. Sci. Adv. 5:eaaw0759. 10.1126/sciadv.aaw0759 31579818 PMC6760924

[B60] Wortman S. E. Francis C. Bernards M. L. Drijber R. A. Lindquist J. L. (2012).Optimizing cover crop benefits with diverse mixtures and an optimizing cover crop benefits with diverse mixtures and an alternative termination method. Agr. J. 104, 1425–1435. 10.2134/agronj2012.0185

[B61] Wu D.-M. Yu Y.-C. Xia L.-Z. Yin S.-X. Yang L.-Z. (2011). Soil fertility indices of citrus orchard land along topographic gradients in the three gorges area of China. Pedosphere 21, 782–792. 10.1016/S1002-0160(11)60182-3

[B62] Ye X. Q. Yan Y. N. Wu M. Yu F. H. (2019). High capacity of nutrient accumulation by invasive solidago canadensis in a coastal grassland. Front. Plant Sci. 10:575. 10.3389/fpls.2019.00575 31134115 PMC6514223

[B63] Zhang Y. Han M. Song M. Tian J. Song B. Hu Y.. (2021). Intercropping with aromatic plants increased the soil organic matter content and changed the microbial community in a pear orchard. Front. Microbiol. 12:616932. 10.3389/fmicb.2021.616932 33643243 PMC7907656

[B64] Zhang Y. Q. Hou L. Y. Li Z. C. Zhao D. X. Song L. G. Shao G. D.. (2020). Leguminous supplementation increases the resilience of soil microbial community and nutrients in Chinese fir plantations. Sci. Tot. Environ. 703:134917. 10.1016/j.scitotenv.2019.134917 31759708

[B65] Zhao Z. Ma Y. Feng T. Kong X. Wang Z. Zheng W.. (2022). Assembly processes of abundant and rare microbial communities in orchard soil under a cover crop at different periods. Geoderma 406:115543. 10.1016/j.geoderma.2021.115543

[B66] Zhou X. Wang J.-T. Wang W.-H. Tsui C. K. M. Cai L. (2021). Changes in bacterial and fungal microbiomes associated with tomatoes of healthy and infected by *Fusarium oxysporum* f. sp. lycopersici. Microbial Ecol. 81, 1004–1017. 10.1007/s00248-020-01535-4 32588072

[B67] Zhou X. Wang J.-T. Zhang Z.-F. Li W. Chen W. Cai L. (2020). Microbiota in the rhizosphere and seed of rice from China, with reference to their transmission and biogeography. Front. Microbiol. 11:995. 10.3389/fmicb.2020.00995 32754120 PMC7365946

