# Supplementary figures and images for: Changes to bacterial communities and soil metabolites in an apple orchard as a legacy effect of different intercropping plants and soil management practices
Source: Front Microbiol. 2022 Aug 8;13:956840. doi: 10.3389/fmicb.2022.956840 (PMC9393497; doi:10.3389/fmicb.2022.956840)

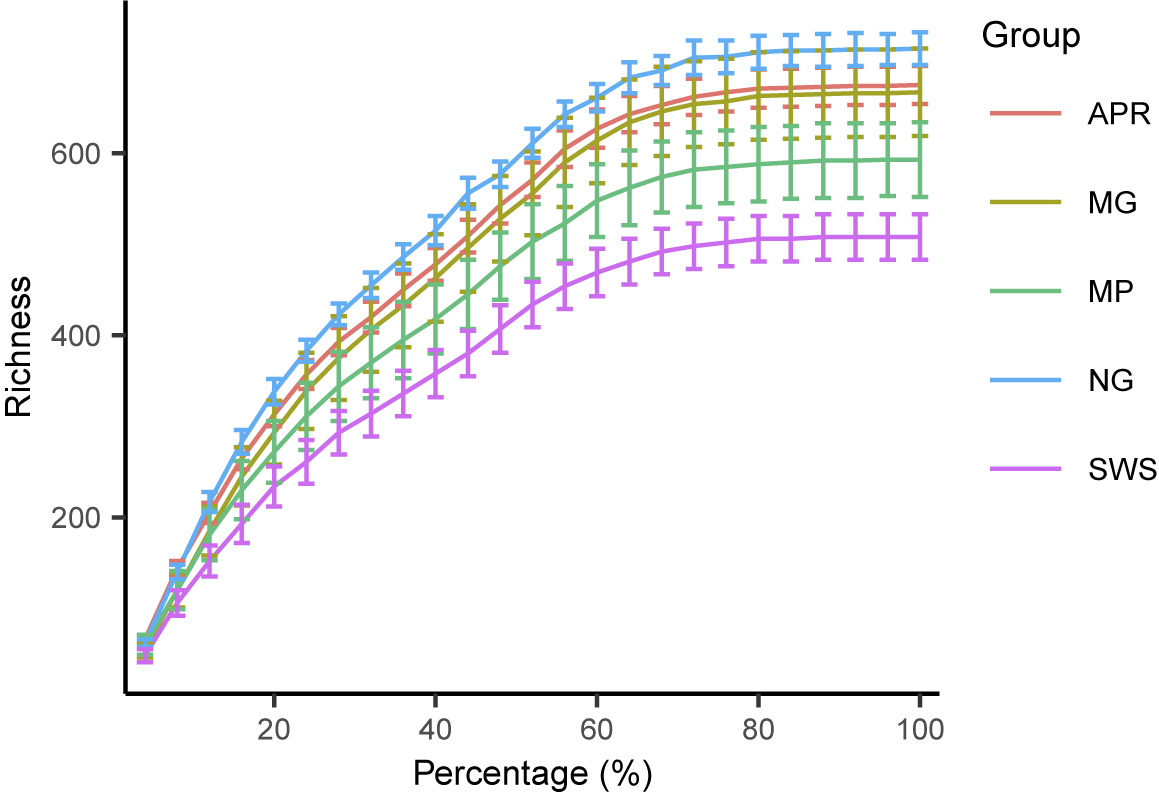

Supplement: Supplementary Figure 1 — Rarefaction curves of bacterial groups from apple, licorice, grass, pepper, and SWS. [file Data_Sheet_1.zip › Supplementary materials/Supplementary Figure 1.tif]

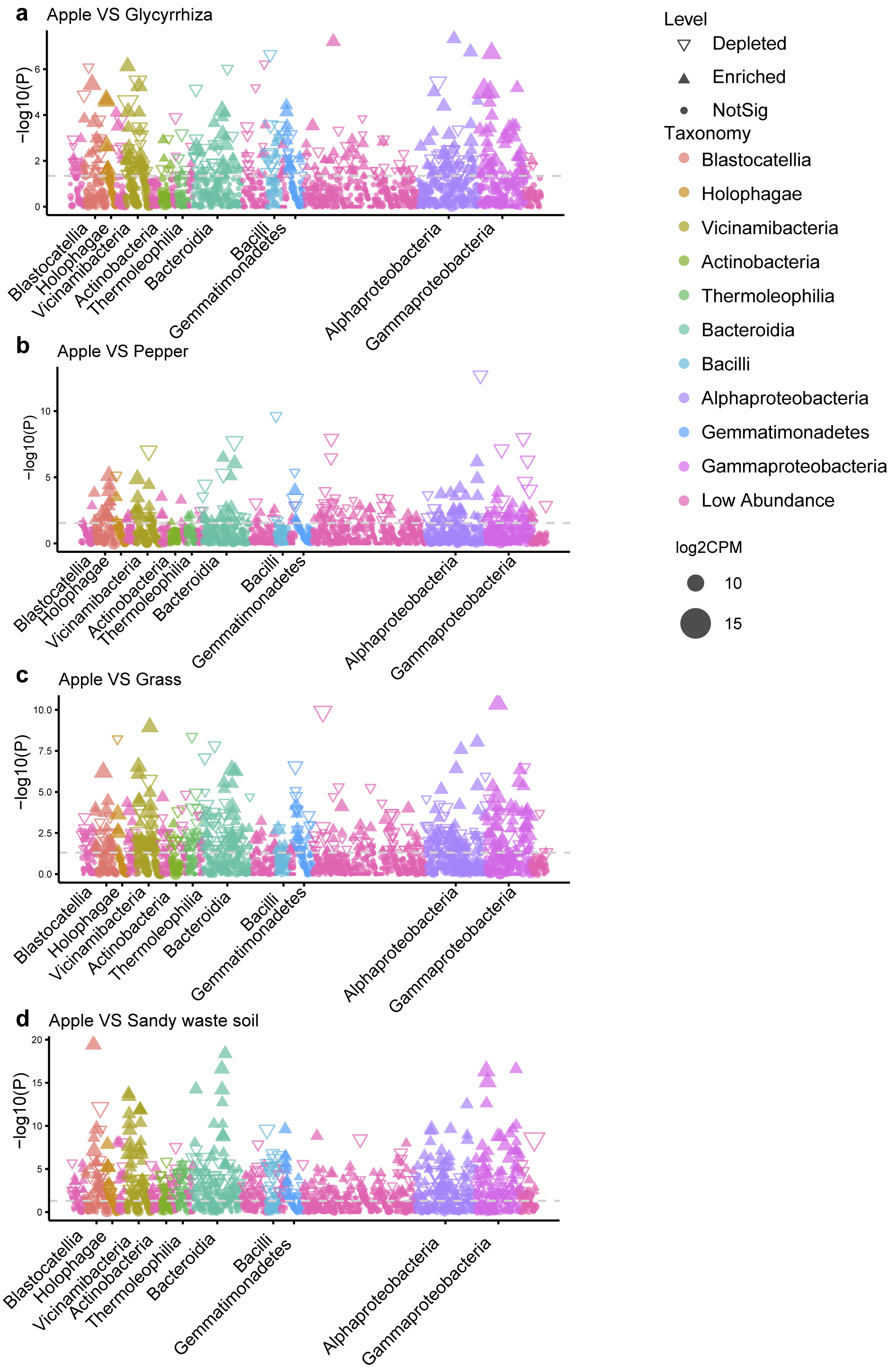

Supplement: Supplementary Figure 1 — Rarefaction curves of bacterial groups from apple, licorice, grass, pepper, and SWS. [file Data_Sheet_1.zip › Supplementary materials/Supplementary Figure 2.tif]

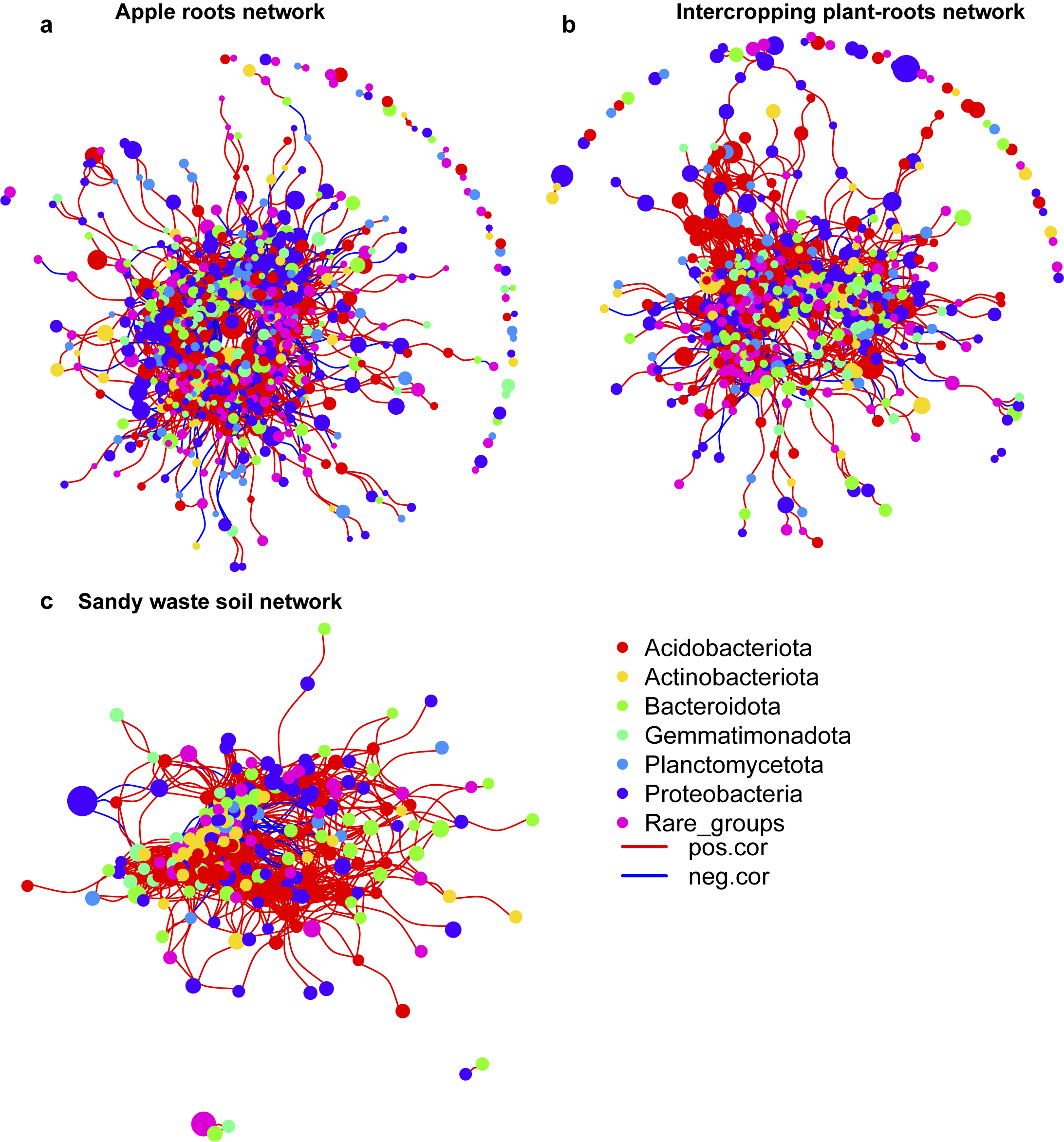

Supplement: Supplementary Figure 1 — Rarefaction curves of bacterial groups from apple, licorice, grass, pepper, and SWS. [file Data_Sheet_1.zip › Supplementary materials/Supplementary Figure 4.tif]

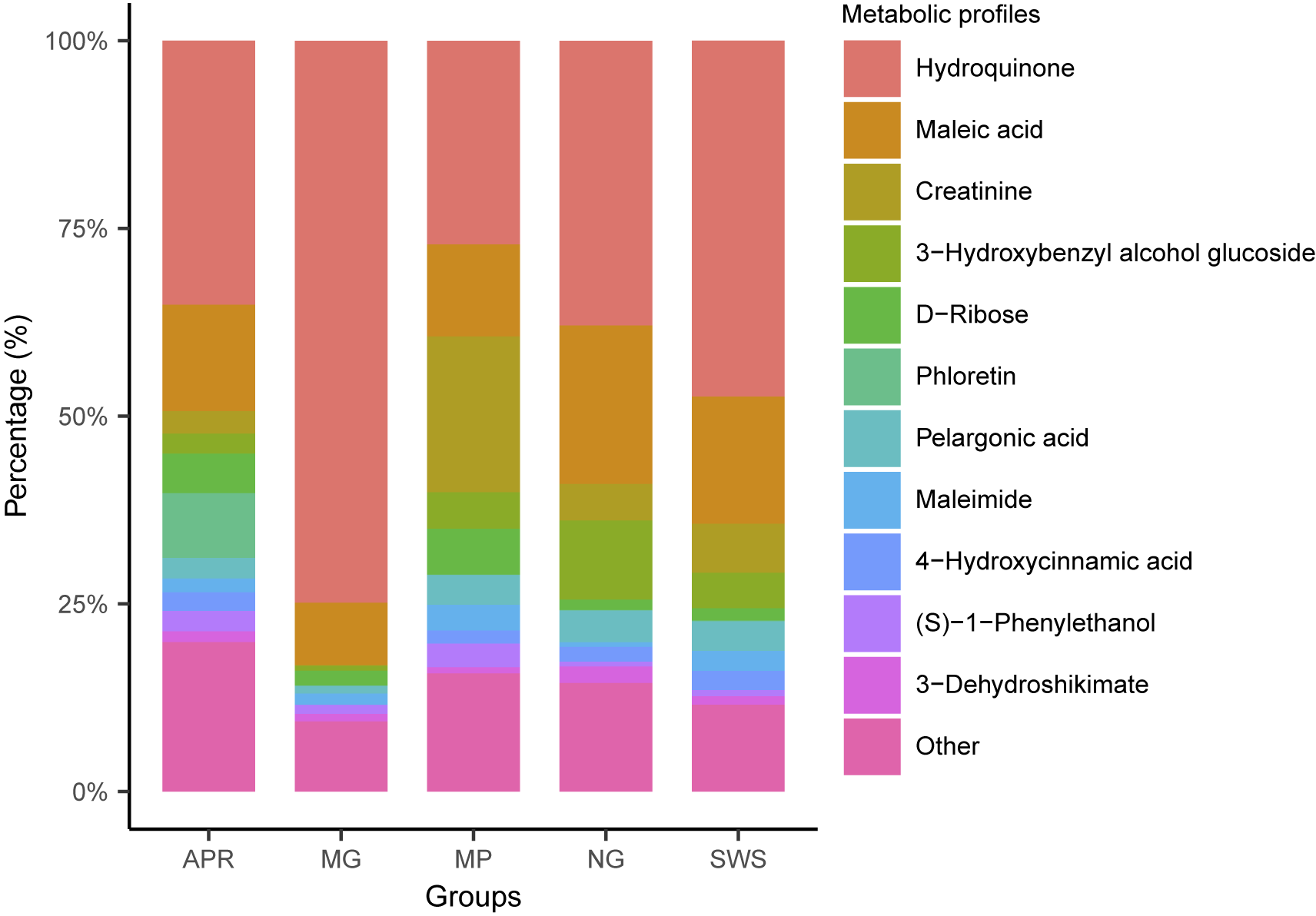

Supplement: Supplementary Figure 1 — Rarefaction curves of bacterial groups from apple, licorice, grass, pepper, and SWS. [file Data_Sheet_1.zip › Supplementary materials/Supplementary Figure 5.tif]

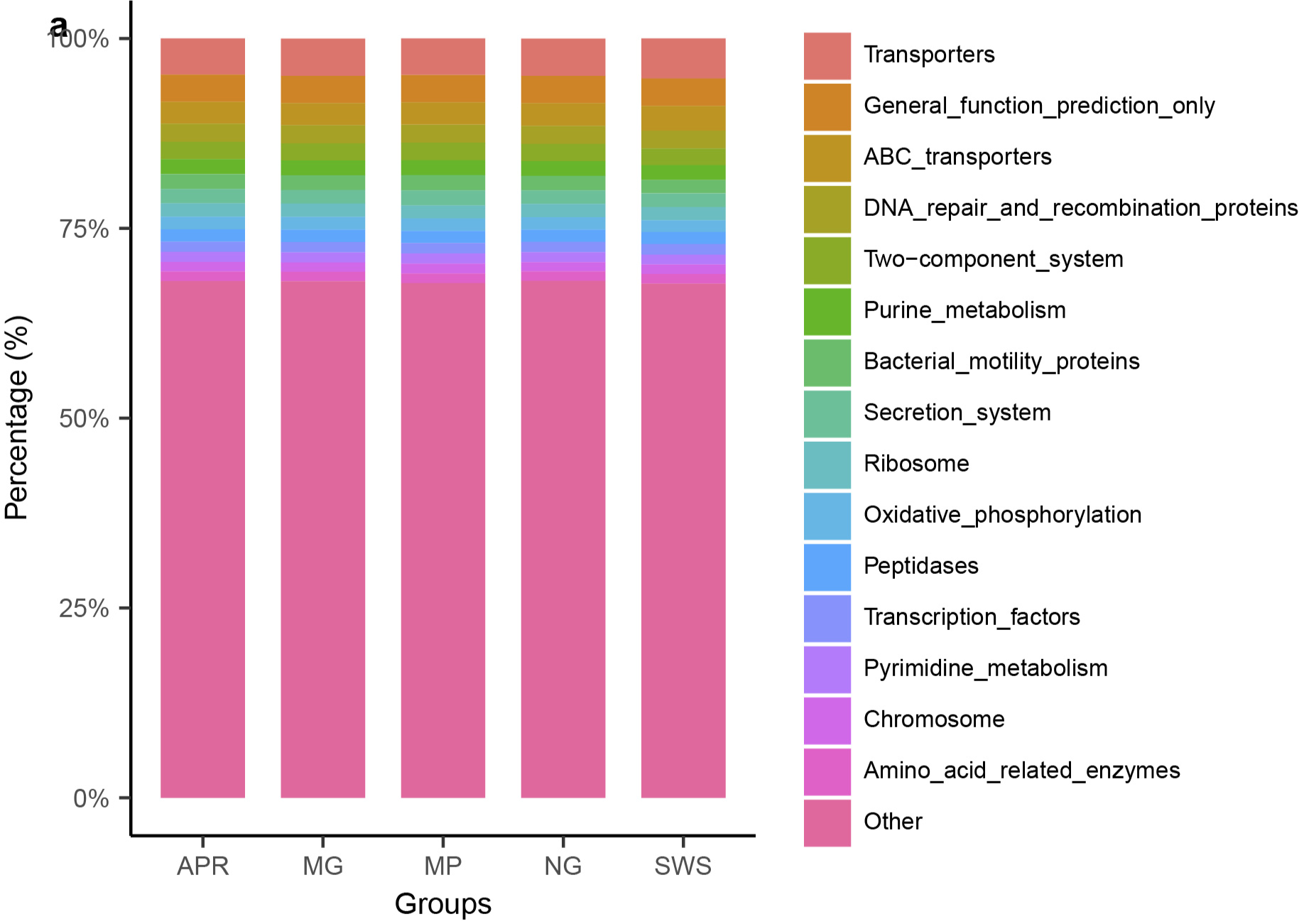

Supplement: Supplementary Figure 1 — Rarefaction curves of bacterial groups from apple, licorice, grass, pepper, and SWS. [file Data_Sheet_1.zip › Supplementary materials/Supplementary Figure 6.tif]

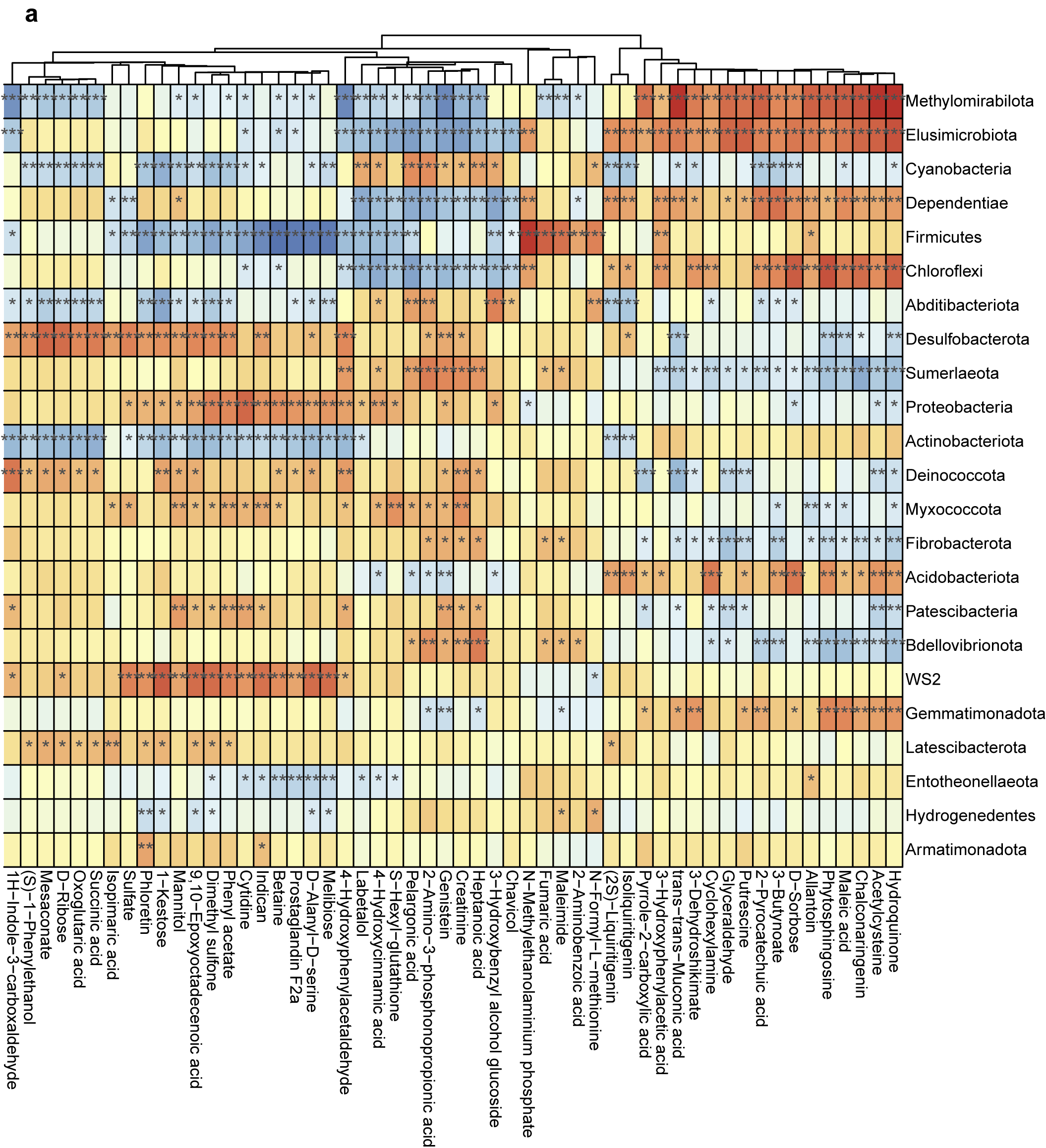

Supplement: Supplementary Figure 1 — Rarefaction curves of bacterial groups from apple, licorice, grass, pepper, and SWS. [file Data_Sheet_1.zip › Supplementary materials/Supplementary Figure 7.tif]

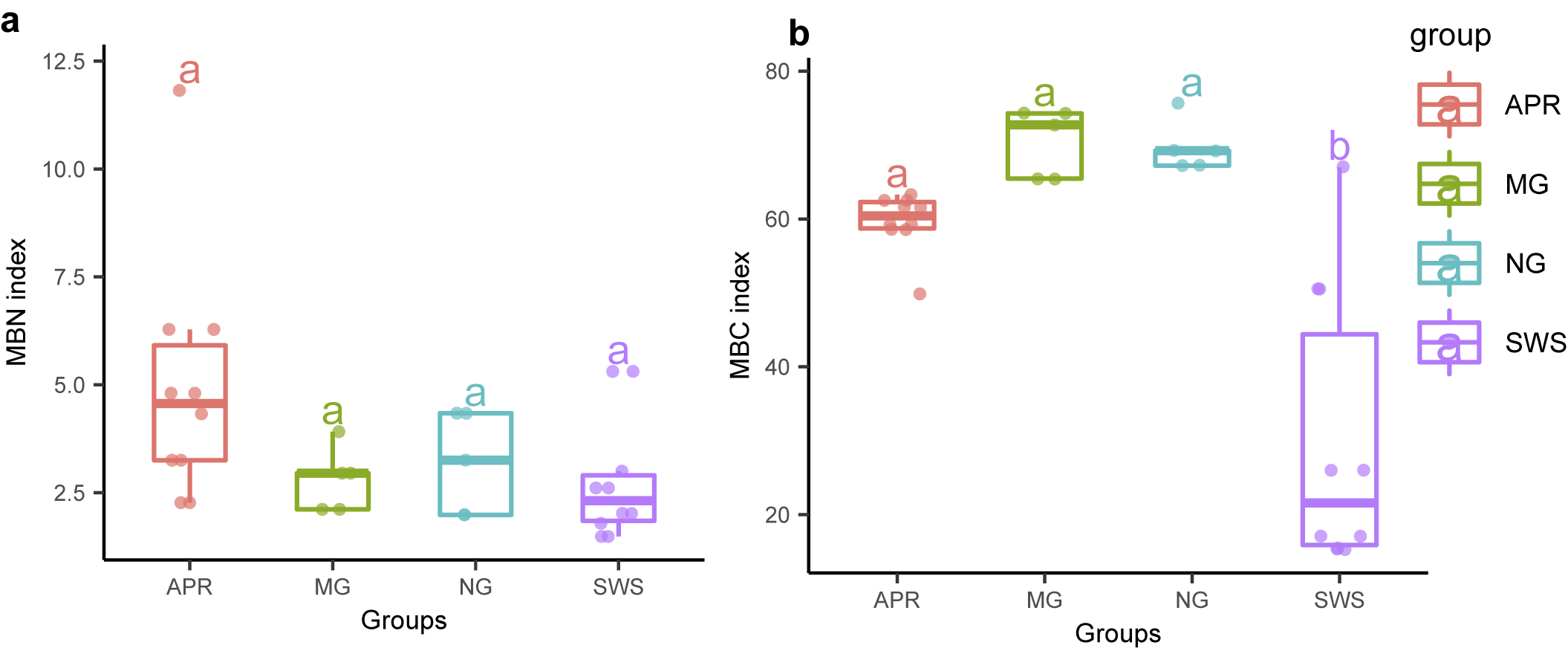

Supplement: Supplementary Figure 1 — Rarefaction curves of bacterial groups from apple, licorice, grass, pepper, and SWS. [file Data_Sheet_1.zip › Supplementary materials/Supplementary Figure 8.tif]
